# Employing hydrogels in tissue engineering approaches to boost conventional cancer-based research and therapies

**DOI:** 10.1039/d1ra00855b

**Published:** 2021-03-12

**Authors:** Javad Esmaeili, Abolfazl Barati, Jafar Ai, Vajihe Taghdiri Nooshabadi, Zeynab Mirzaei

**Affiliations:** Department of Chemical Engineering, Faculty of Engineering, Arak University Arak Iran Ja_esmaeili@yahoo.com; Department of Tissue Engineering, TISSUEHUB CO. Tehran Iran; Department of Tissue Engineering and Applied Cell Sciences, School of Advanced Medical Technologies, Tehran University of Medical Sciences Tehran 14177-55469 Iran; Nervous System Stem Cells Research Center, Semnan University of Medical Sciences Semnan Iran; Faculty of Biomedical Engineering, Amirkabir University of Technology Hafez str. 424 Tehran Iran

## Abstract

Cancer is a complicated disease that involves the efforts of researchers to introduce and investigate novel successful treatments. Traditional cancer therapy approaches, especially chemotherapy, are prone to possible systemic side effects, such as the dysfunction of liver or kidney, neurological side effects and a decrease of bone marrow activity. Hydrogels, along with tissue engineering techniques, provide tremendous potential for scientists to overcome these issues through the release of drugs at the site of tumor. Hydrogels demonstrated competency as potent and stimulus-sensitive drug delivery systems for tumor removal, which is attributed to their unique features, including high water content, biocompatibility, and biodegradability. In addition, hydrogels have gained more attention as 3D models for easier and faster screening of cancer and tumors due to their potential in mimicking the extracellular matrix. Hydrogels as a reservoir can be loaded by an effective dosage of chemotherapeutic agents, and then deliver them to targets. In comparison to conventional procedures, hydrogels considerably decreased the total cost, duration of research, and treatment time. This study provides a general look into the potential role of hydrogels as a powerful tool to augment cancer studies for better analysis of cancerous cell functions, cell survival, angiogenesis, metastasis, and drug screening. Moreover, the upstanding application of drug delivery systems related to the hydrogel in order to sustain the release of desired drugs in the tumor cell-site were explored.

## Abstract 2

Hydrogels and tissue engineering showed their potential in facinating research and developement in cancer therapy.

## Graphical abstract

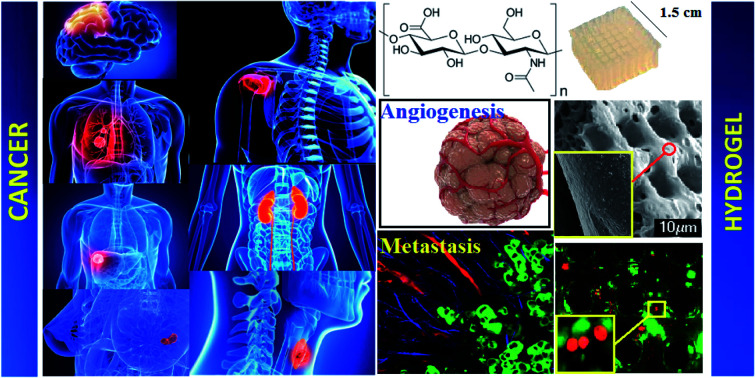

## Introduction

Cancer, as a genetic disease, is introduced as a principle and critical worldwide public health issue, and is the second leading cause of death in the United States.^1^ It is one of the fatal diseases, which appears in different organs, including the breast, bladder, liver, prostate and kidney. Surgery, radiation therapy, chemotherapy, immunotherapy, targeted therapy, hormone therapy, stem cell transplant, and precision medicine are standard cancer treatments, which are generally applied for patients suffering from cancer.^2^

**Fig. 1:**
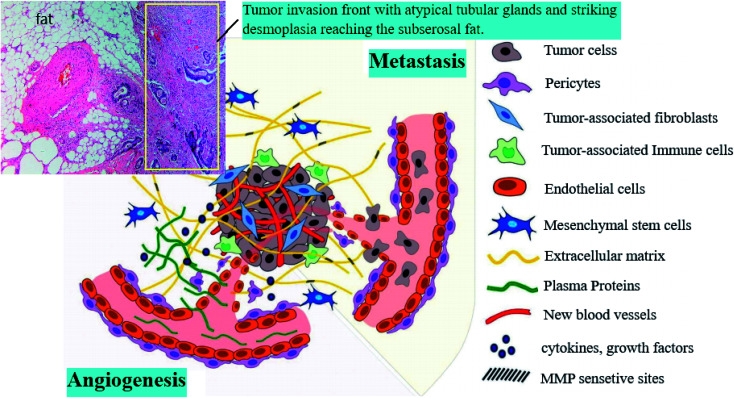
Components of the tumor microenvironment.^3^

The proliferation of abnormal cells and metastasis are the vital stages of cancer progression, which result in cancer relapse. The cancer microenvironment stroma includes many distinct cells, such as blood and lymphatic endothelial cells, epithelial cells, mesenchymal stem cells, pericytes and fibroblasts (Fig. 1).^3,4^ Endothelial cells, macrophages, or cancer-associated fibroblasts (CAF) as tumor microenvironment (TME) cells sustain proliferation and migration within the environment by dynamic interactions with cancer cells.^5^

The mortality and morbidity of cancer have been rising even by ignoring the risk factors, such as being overweight, smoking and aging, without a proper diet and growth of population.^6^ Researchers all over the world have been trying to find out an ideal therapy for distinct types of cancers. In some cases, they have achieved excellent results, but more efforts and studies must be done.^7^ Different studies have led to discovering various methods and drugs for cancer therapy. However, clinical trials are expensive and are often slowed down by high failure rates due to misguided preclinical models. Therefore, more preclinical investigations are still needed to reach the exact prediction and drug diagnosis.^6^

In some cases, cancer is healed just by surgery. In other cases, healing is done by employing chemotherapy (CT), radiotherapy (RT) as adjuvant therapies, and also molecular tools.^1^ For instance, mastectomy and breast-conserving therapy are well-employed as local therapies for the first stage of invasive breast cancer.^2,8^

The initial CT is known as induction CT before the local chemotherapy. By combining this method with other treatments, noticeable advantages are achieved, including the reduction of primary tumors to improve the local regional control, and also the reduction of the metastatic relapsing risk.^9^ However, there are some side effects as well, including gastrointestinal, musculoskeletal or constitutional symptoms, loss of appetite, vomiting, constipation or diarrhea, nausea, and the most common and uncomfortable one is hair fall.^10^

Still, surgery and chemo and radiotherapies are the most common methods to fight cancer, but scientists have introduced a new structure named hydrogel. Hydrogels are hydrophilic gels, and are known as an incomparable class of three dimensional (3D) and cross-linked polymeric networks. They can retain a large amount of water, aqueous solvents, and biological fluids within their structures, which are not dissolved in water.^11^ Hydrogels are prepared through covalent and/or noncovalent interactions, and with the transverse connections of monomeric or polymeric networks and chains.^12^ Functional groups attached to the polymeric backbone play a leading role in absorbing water, and cross-links between the polymeric chains are responsible for resistance against dissolving in water. Mainly, there are two types of hydrogels, including synthetic and natural hydrogels. The natural type can be employed in biological applications. They can be used as media to retain cells or as a reservoir of drugs, and it is challenging to control their mechanical properties. Besides, because of their high water content, they possess a degree of flexibility, which is very advantageous due to their similarity to natural tissues.^13^ The synthetic hydrogels mostly show well-defined structures and reproducibility. Hydrogels also can be classified based on other different parameters, including the source, configuration (amorphous, semi-crystalline and crystalline), polymeric composition (homopolymeric, copolymeric and multipolymer interpenetration), cross-linking type (chemical, ionic and physical), physical appearance, electrical charge of the network (cationic, anionic and nonionic), and amphoteric electrolyte (both acidic and basic groups).^14–16^

Hydrogels are considered the main tools in cancer therapy through various approaches, including Drug Delivery Systems (DDSs),^17,18^ Smart Hydrogels (SH),^19,20^ Hydrogel-Based Implants,^21^ Organ-On-a-Chip (OOC),^22^ 3D models (3DM),^23^ 3D Printing (3DP),^18,24^ and a combination of the above ^25^ (Table 1). Thanks to the extraordinary characteristics of hydrogels, many attempts have been made for tumor diagnosis, tumor modeling, anti-cancer drug screening, and cancer therapy.

**Table 1:** A summary of hydrogel applications in cancer therapy

Type of cancer	Cell	Polymer (hydrogel)	approach	subject under study	Drug	Reference	Year
Breast	Stromal cell MCF-7 cells	Gelatin collagen	3D model in vitro	Behaviour of breast cancer cells in response to different environmental stimuli	—	30	2015
Breast	Human umbilical vein endothelial cells (HUVECs)	Carbon dots, polyethylene glycol, folic acid	Organ-on-chip	To rapidly screen drugs and drug delivery systems	Doxorubicin (DOX)	31	2018
Breast	MCF-7	PEGylated fluorocarbon nanoparticles, PEGylated hydrocarbon nanoparticles	Drug delivery system	Simultaneous and segregated delivery of multiple drugs	PTX-DOX	32	2015
Skin	Oral squamous cell carcinoma (D20 and Cal27 cells)	Collagen	3D model *in vitro*	Generate tissue-engineered models and compared with patient biopsies	—	33	2011
Melanoma	Cell lines. B16.F10 mouse melanoma cells	Matrigel	3D tissue model (rotating wall vessel bioreactor system)	Cell interactions and collection of new information about cancer development	—	34	2009
Prostate	M12 and LNCaP C4-2	poly(2-hydroxyethyl methacrylate)	Hydrogel	Describe a capillary force-based method for seeding the human prostate cancer cell lines	—	35	2013
Prostate	Human PCa cells (LNCaP, C4-2, and C4-2B)	Matrigel matrix and chitosan–alginate	3D model *in vitro*	A new matrix for studying prostate cancer cell-lymphocyte interactions *in vitro*	—	36	2012
Liver	HepG2 (human HCC cell line)	Co-polyester polymer (Zimmer and Peacock,92000042)	3D printing	Real-time immunodetection of liver cancer cells	—	37	2017
Liver	Human hepatocellular carcinoma cells (FLC-4 cells)	Tetra ethoxysilane, poly- dimethylsiloxane, galactosylated silk fibroin -based, collagen	3D scaffold	A hepatic tissue engineering application of three-dimensional (3D) poroussponges	—	38	2011
Brain	Glioma (D54 and D54-GFP-luc) cell lines	Chitosan alginate	Hydrogel (*in vivo*)	Injectable hydrogels for localized chemotherapy and radiotherapy in brain tumors	—	39	2018
Brain	Glioma stem cell	Gelatin/alginate/fibrinogen	3D bioprinting *in vitro*	Glioma genesis and drug resistance	—	40	2016
Lung	H1975 human NSCLC adenocarcinoma cells, human primary airway epithelial cells, human lung microvascular endothelial cells	Poly-dimethylsiloxane (PDMS), gelatin, polyester (polyethylene terephthalate [PET])	Organ-on-chip	To create *in vitro* human orthotopic models of non-small-cell lung cancer	Tyrosine kinase inhibitor	41	2017
Bladder	Fibroblasts and urothelial cells (RT4 (NMIBC) and T24 (MIBC) cell lines)	Gelatin collagen	3D model *in vitro*	Reconstitution of the microenvironment	—	42	2017
Glioblastoma	Astrocytes	Lauroyl-gemcitabine lipid (GemC12-LNC)	Nanodelivery of drugs	Drug combination using an injectable nanomedicine hydrogel for glioblastoma treatment	Paclitaxel (PTX)	65	2019
Solid tumors	HeLa cells	mPECT(D)/GDDC-4(R)/α-CD	Drug delivery system	Co-delivery of drugs and siRNA	GDDC-4/siRNA	66	2019
Prostate	DU-145 cells and PC-3 cells	Two zinc ions (ZIs)-responsive short peptide dendrons (E3FID and E3FNP) hydrogels	Drug delivery system	Conjugation of forky peptides and nonsteroidal anti-inflammatory drugs (NSAID) self-assemble into supramolecular hydrogels	Docetaxel (DTX)	70	2019
Head and neck	HSC-3 cells	Self-assembling peptide	Drug delivery system	Local Co-delivery of drugs	Doxorubicin and curcumin	79	2019
Breast	TNBCs	RNA-triple helix	Drug delivery system	Incorporating the RNA-triple-helix and siRNA duplexes of CXCR4 into the same RNA nanoparticles	miRNA-205, miRNA-221	111	2020
Breast	MCF-7 cells	Hyaluronic acid	3D model *in vitro*	Mimicking extracellular matrix	—	170	2019

The rheological properties of hydrogels are also important for developing efficient hydrogel systems by controlling their physical and mechanical properties. The swelling and mechanical behavior of hydrogels can be characterized by rheological determinations. These rheological and mechanical properties are significantly important towards understanding cellular mechanotransduction and the behavior of delivery systems. Bio hydrogels are known by their viscoelastic properties with a modulus of a few kPa, which is much smaller than the moduli of other solid materials. The rheological determination of hydrogels may be challenging because of their soft nature. The results of the studies indicate that the rheological characteristics of hydrogels can be strongly controlled by different parameters, including the chemical composition, polymer concentration, density of cross linkers, degree of substitution, initiator concentration and irradiation conditions. In rheology experiments, the frequency of the oscillation determines the flow condition by calculating the elastic and viscous behavior. The increase in the oscillatory frequency and concentration of the polymers steadily increases the elastic and viscous moduli.^26,27^

In this paper, we review how hydrogels can boost and elevate conventional cancer therapies. Besides, we outline information about using hydrogels for tumor removal, and the significant role of hydrogels in the TME 3D models and metastasis. At the end, we state some questions with vague answers, which require more studies and experiments.

## Hydrogels

Hydrogels (Hydrophilic gels that are usually referred to as Hydrogels) are known as an incomparable class of three dimensional (3D) and cross-linked polymeric networks that can retain a large amount of water, aqueous solvents, and biological fluids within their structures, while not dissolving in water. A hydrogel with the transverse connection of monomer or polymer networks or chains is produced through covalent and/or noncovalent interactions.^12^ The functional groups attached to the polymeric backbone play a leading role in absorbing water, and the cross-links between the polymeric chains are responsible for resistance against dissolving in water. There are two kinds of hydrogels: synthetic and natural. The natural type can be employed for biological purposes, like media to retain cells or as a reservoir of drugs, while showing weak challenges in controlling their mechanical properties. Moreover, they possess a degree of flexibility that is very similar to natural tissue due to their high water content. The synthetic hydrogels mostly show well-defined structures and reproducibility with high mechanical properties.^28^ Hydrogels are generally classified based on the source, configuration (amorphous; non-crystalline; semi-crystalline: a mixture of amorphous and crystalline phases; crystalline), polymeric composition (homopolymeric; copolymeric; multipolymer interpenetrating polymeric), cross-linking type, physical appearance electrical charge of network (cationic or anionic); nonionic; amphoteric electrolyte (both acidic and basic groups); and zwitterionic (polybetaines; both anionic groups and cationic groups in each structural repeating unit). In general, to fabricate a hydrogel with the appropriate mechanical, physical, chemical and biological features, a combination of both synthetic and natural polymers is necessary. In this case, scientists try to optimize the final combination.^29^

## Programmable hydrogels

These unique hydrogels are intelligent substances with notable applications, which can be changed according to how polymer structures are responsive to the environmental provocation,^43^ and both internal and external factors.^19,44^ Recent progress in both natural and synthetic polymeric substances have led to the development of different sensitive hydrogels to three categories of simulations, including: biological (DNA and aptamer, protein and peptide, enzymes and small metabolite), chemical (redox, ions and pH^12,45^) and physical (temperature,^46^ light,^47^ and electric field^48,49^) stimulations. The polysaccharides, like carboxymethylcellulose, starch, alginate, chitosan, and carrageenan, are suitable and mostly employed as candidates to develop hydrogels as biomaterials. This is due to their exciting properties, including non-toxicity, biocompatibility, biodegradability, and low-cost.^50,51^ Many research studies were done to employ hydrogels as smart or programmable materials to remove tumors by appropriate strategies.^43^ Some hydrogels proved their capability of providing a sustained release of the chemotherapeutic drugs, and effective treatment for cancer therapy.^52^ Furthermore, low molecular-weight hydrogels have already shown promise to be an accurate candidate for the sustained release of chemotherapeutic drugs.^53^ Hydrogels are also used as cell reservoirs to encapsulate therapeutic cells due to their features of controllable structures, high porosity, and physicochemical characteristics.^54^ The main drawback of the standard treatments is their side effects. In contrast, *via* controllable hydrogels, anti-tumor agents not only can be developed, but also can attack cancerous cells without killing the healthy cells. It was approved that toxic anti-cancer drug-associated polymers significantly decreased side effects.^55^

Scientists have studied the brain tumor Glioblastoma (GBM) as a cancer model for cell therapy by designing an injectable hydrogel to localize CT and RT.^39^ A programmable chitosan hydrogel loaded with Temozolomide (TMZ) capable of releasing chemotherapy drugs, while retaining radioactive isotopes agents, was employed for centralizing radiation and chemotherapy within the surgical cavity. Developing an injectable hydrogel as a smart system, which can fill the 3D volume of the surgical cavity, delivers a homogeneous radiotherapeutic dose to all areas of the hole (including additional targeted margin) without considering the form and shape of the cavity, and the local delivering of chemotherapeutic agents.^39,56^

The migration and relocation of cancer cells through the blood-brain barrier (BBB) is a crucial process of brain metastasis. The BBB plays a crucial role in maintaining and keeping drug delivery to the brain, and also brain homeostasis.^57^ An *in vitro* reliable and robust model, which can be translated to the *in vivo* situation and reflects the healthy and diseased state of BBB, is necessary. According to the research of Sanati-Nezhad and his colleagues,^58^ biomimetic models are fundamental to expand the diseased models of the BBB, anticipating the permeability of the molecules across the BBB (in both healthy and unhealthy states), determining and controlling the efficiency of drug delivery across the BBB into the brain tissue, measuring the removal mechanisms of the wrong binding proteins from the brain, and assessing the contribution of the BBB in the progress and improvement of neurodegenerative diseases or brain-tumor upon the unison with biomimetic brain tissues.^58^

**Fig. 2:**
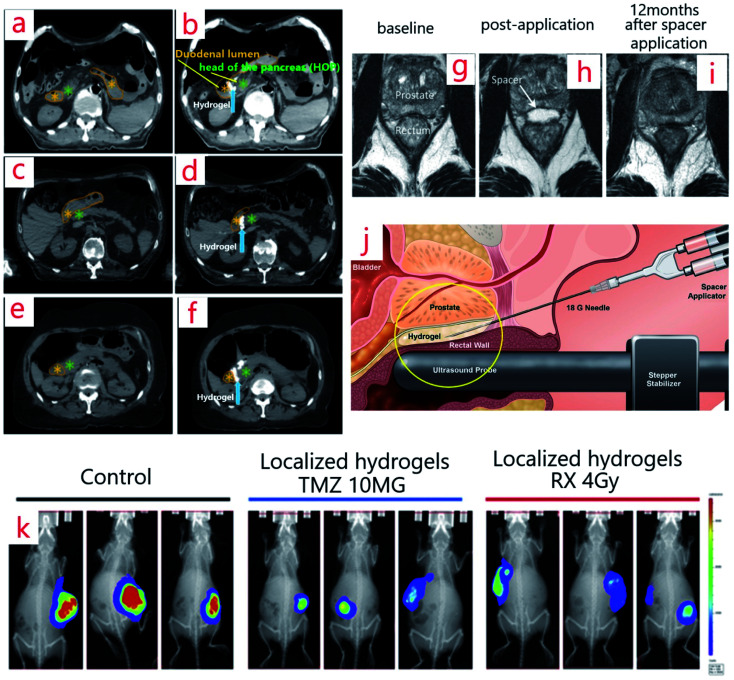
(a–f) Computed tomography scans (CTS) before (a, c, e) and after (b, d, f) hydrogel spacer injection between the HOP and duodenum.^67^ (g–i) MRI image of a spacer patient at baseline (g), post-application (h), and 12 months after spacer application (i).^65^ (j) The way of spacer injection (PEG hydrogel spacer injection) between the prostate and rectal wall.^59^ (k) Bioluminescent imaging (BLI) images of three mice implanted with radiotherapy hydrogels (right), chemotherapy (middle), control (left) at day 27 post-implantation.^39^

Interestingly hydrogels can quickly be injected by syringe (Fig. 2j).^59^ A significant and noticeable decrease in the rectal dose is attainable by using the spacer.^60,61^ If a spacer is injected between the anterior rectal wall and prostate, then the rectal wall is protected. Thus, in this case, the hydrogel spacer is a successful candidate for making a gap between the rectum and prostate in prostate cancer treatment.^62–64^ Those patients who used the spacer received more doses of drugs by the prostate (Fig. 2g–i).^65^ To improve the efficiency of the spacers, long time clinical results are necessary. So, in 2010–2011, scientists employed a biodegradable hydrogel-based spacer (as a smart hydrogel) for 114 patients who received RT to the prostate, and they were under study for 5 years. The results revealed significant and excellent treatment tolerability, especially considering bowel problems.^66^ Similarly, similar spacers can be employed for other types of cancers. Using the Novel Absorbable Radiopaque Hydrogel Spacer for pancreas cancer was approved by increasing the space between the duodenum and the head of the pancreas (HOP) in 3 human cadaveric models (Fig. 2a–f).^67^

An injectable chemo-radio-hydrogel implant can modify the local control, effectiveness, and final result of the poor diagnosis of tumors. Fig. 2k shows the bioluminescent images (BLI) of three mice implanted with the smart chitosan-based hydrogel (right), chemotherapy (middle), and control (left) on day 27 post-implantation. Following localized treatment, a decline in the tumor size in the arms can be seen in contrast with that of the control group.^39^

Researchers prepared shear-thinning injectable magnetic hydrogels (PEGylated Fe_3_O_4_ nano-particles and α-Cyclodextrins: α-CD), and proved their therapeutic and remedial potential to remove the postoperative relapsing of the (breast) tumor with mild, gentle synthesis, and least invasive injection *in vivo*.^68^ They believed that the hydrogel and its features could be manipulated. The concentration of the doxorubicin (DOX) drug affected the hydrogel properties significantly. The higher level of the drug caused a lower viscosity of hydrogel, and resulted in affecting the drug release (DR).^69^ The Magnetic Supramolecular Hydrogel (MSH) revealed the magnetocaloric liquid-conformal ability to completely match and fill the irregular cavity after tumor removal without a blind angle when it goes under an intermittent current magnetic field. MSH synergistically demolishes the tumor and hinders the local relapse entirely of breast cancer following surgery.^68^ The dual and ambivalent structure of the MSH caused a distinct release of different drugs from a unitary sample. In addition, the long-term local delivery of MSH synergistically omits the tumor, and stops the local recurrence of breast cancer after surgical resection. Moreover, MSH with the controlled administration of combined thermo-chemotherapy demonstrated noticeable superiority, considering the hampering of postoperative cancer recurrence.^68^

Bio-responsive hydrogels, as another form of programmable hydrogels, can be loaded by agents or receptors, and be changed in the case of any external stimuli. Here, there is a particular interest to develop autonomous hydrogel-based systems to detect cancer markers, and respond to them to repair the cancerous area or tumor removal.^70^ As we know, modified hydrogels can contain small biomolecules that selectively attach or bind to bio-macromolecules, such as antibodies or protein receptors. Also, enzymes are strongly selective. Therefore, hydrogels can be programmed to respond to a particular enzyme by integrating the specific substrate.^71^

Based on our studies, some of the tumors may resist a single drug. This is due to the heterogenetic nature of the tumor and malignant cells, and it is necessary to employ more therapeutic agents concurrently. To deliver multiple drugs with various molecular targets in the cancerous area, programmable hydrogels are proper candidates to prevent drug resistance and overcome tumor metastasis.^72^ A synergistic therapy that combines drug/siRNA to remove the tumor was revealed to have tremendous popularity for enhancing the antitumor efficiency and overcoming the side effects. In one study, DOX and siBcl-2 were selected as a co-delivery system for synergistic cancer therapy by employing a supramolecular hydrogel based on nanoparticles.^73^ Therapeutic agent delivery is carried out in a continuous or discontinuous manner. As programmable systems, hydrogel-based DDS results in more effective drug delivery compared to the conventional systemic therapies. This is because the hydrogel-based DDS provides a sustained and high-dose release of the chemotherapeutics at the tumor site. Norouzi *et al.* reviewed this aspect by focusing on the injectable hydrogel-based DDS for local cancer therapy.^74^ A steady and uniform dose delivery of the chemotherapeutic agents leads to lower cancerous cell elimination, in contrast to a short-term, but high-dose delivery. Hydrogels can be engineered in a way to provide a high-dose drug release in a short period, or lead to a stable distribution of therapeutic agent, but for a long time.

Anti-tumor drugs can be injected or transported directly to the cancer microenvironment, but it entails engineering a smart hydrogel to act as an anti-tumor agent without inflammation.^75^ According to the previous studies, inflammation is known as a critical and vital component of tumor progression.^76^ Anti-inflammatory drugs conjugated with one or more anti-tumor agents can be loaded into a hydrogel to prevent cancer cell proliferation.^77^

### Hydrogels for cancer drug delivery systems

The restricted impact of common drugs has provoked scientists to develop more new ways of drug delivery. DDS is known as a system to provide the controlled delivery of therapeutic agents, proteins and vitamins to a certain spot of the body, and boost the quality of delivery by controlling the exact time and location. DDSs are categorized as the parenteral pulmonary, transdermal, oral, cardiovascular, or nasal systems. No drug can perform and be active with one hundred percent efficiency. By inserting the drugs into the body, just a limited dose of it is impressive. This is due to the precipitation in nontarget tissues and excretion from the body. This is why the drugs have off-target side effects. As for the disadvantages of common medication intake, it can be referred to as low efficiency, high side effect, and high dosage of drug per short time, weak metabolism, pain, less targeting, and high cost. However, according to our recent survey, the hydrogel-based DDS can be more efficient than standard therapies in clinics.

By taking cancer therapy into account, delivery systems can be under control. It can also be targeted because the therapeutic agent delivery to cancerous tissues is a significant issue, owing to the dynamic complexity of TME. Therefore, the desire to reach systemic pharmaceutical therapy (DDS) has increased. The novel DDS includes the improvement of the amount of pharmaceutical agent loading in drug carriers, cellular receiving of drug carriers, and the sustained release of drugs. Many research studies are focusing on different types of cancer. The mentioned studies in the previous section can be considered as one aspect of DDSs. In the following section, more information is provided about CDDS (Table 2). In the rest of this review, based on what we inferred from the literature studies, a review of the hydrogel-based DDS for cancer therapy is presented based on its sensitivity and targeting.

**Table 2:** Employed sensitive hydrogels for cancer drug delivery systems^a^

Hydrogel	Drug	Sensitivity	Cancer	Reference
PPZ	Silibinin	Thermosensitive	Colon	107
PEG-FA, PPLL	ME, 5-FU	pH-sensitive	Colon	108
PLGA-PEG-PLGA/CND	DOX	Thermosensitive	Prostate	109
Dgel AuNP	DOX	Photosensitive	Melanoma	110
PEG-DMA	TMZ	Photosensitive	Glioblastoma	111
Poloxamer P407, Poloxamer P188	PTX	Thermosensitive	Pancreatic	112
Akt1 shRNA, PTX	CLA-coupled poloxamer/PEI-*alt*-PEG	Thermosensitive	Breast	113
PEG NPs	DOX, curcumin	pH-sensitive	Hepatocellular carcinoma	87
PEG-PCL-PEG/MPEG-PCL	DDP, PTX	Thermosensitive	Lung	114
P407, P188, graphene oxide/CS	DTX	pH/temperature	Sarcoma	115

a poly(organophosphazene): PPZ; aldehyde-functionalized four-arm PEG: PEG-FA; 4-arm PEG-*b*-poly(lysine): PPLL; metformin: ME; 5-fluorouracil: 5-FU; poly(lactide-*co*-glycolide): PLGA; polyethylene glycol: PEG; clay nano-disk: CND; paclitaxel: PTX; conjugated linolenic acid: CLA; poloxamer 407: P407; poloxamer 188: P188; polyethylenimine – PEG diacrylate: PEI-*alt*-PEG; PEG nanoparticles: PEG NPs; PEG-poly(3-caprolactone)-PEG: PEG-PCL-PEG; monomethoxy PEG-poly(3-caprolactone): MPEG-PCL; DNA hydrogel: Dgel; gold nanoparticles: AuNP; temozolomide: TMZ; PEG dimethacrylate: PEG-DMA; chitosan: CS.

#### Targeting

DDSs have been introduced as a potent system to augment the pharmacological and therapeutic characteristics of drugs.^78^ This system not only decreases the side effects of drugs, but also supplies a sustained release of drugs at the tumor site. An impressive targeted DDS comprises retain, evade, target, and release.^79^ Drug targeting strategies are divided into categories of *active* and *passive*. Nanoparticles or microspheres have been being widely under study for cancer therapy, but due to their shortcomings, there has been a surge in interest for *in situ* hydrogels.^80^

Combining one or more biologics (*e.g.*, other monoclonal antibodies (mAbs), nucleic acids or proteins) with an antibody leads to tremendous clinical advantages. Antibody-drug conjugates (ADCs) are a promising type of potent biopharmaceutical agents engineered as a targeted remedy for cancer treatment.^81^ Targeted cancer therapy with mAbs holds high promise in killing cancer cells without the systemic toxicity from the chemotherapeutic agents. ADCs incorporate the cancerous cell-killing power of a cytotoxic therapeutic agent with the targeting ability of an antibody. In consideration of this, ADCs-loaded hydrogels are potent candidates for tumor removal. A hydrogel for the sustained and stable release of pharmaceutically relevant mAbs was introduced as a robust vehicle. Silk hydrogel as a delivery matrix for the slow and gradual release of the loaded drugs/proteins are proper and appropriate for the long-term sustained release of chemotherapeutic agents.^82^ Similar to ADCs, antibody-nanoparticle conjugates (ANCs) can be engineered to receive various pharmacological properties and be employed for cancer treatment. In the context of CDDs, a few studies have reported the combination of hydrogels and ANCs aimed toward cancer therapy by decoration the hydrogel with ligands to connect to a target molecule. We believe that this combination will provide the means to reach highly targeted drug delivery.^83^

Angiogenesis and tumor resistance against therapeutic agents (*e.g.*, DOX) are significant viewpoints of cancerous diseases. It has been shown that the chitosan hydrogel may be a potential candidate as a new local carrier for drugs (*e.g.*, fibroblast growth factor and paclitaxel) to target vascularization and control angiogenesis.^84^ A DOX-loaded hydrogel made of chitosan and polyvinyl alcohol was studied to investigate its anti-cancer potential by monitoring the angiogenesis. The results demonstrated by the hydrogel with 1 mg ml^−1^ of DOX depicted a significant reduction in angiogenesis.^85^

To overcome the tumor resistance against therapeutic agents, hydrogels are engineered for multi-drug delivery. In one study, a self-assembling peptide hydrogel loaded with DOX and curcumin was employed for neck and head cancer.^86^ This engineered hydrogel not only provided local dual drug delivery, but also overcame tumor resistance against the released drug. Sometimes, more than one target must be considered in engineering a DDS. A dual-targeting hydrogel conjugated with an estrone (ES) and RGD (Arg–Gly–Asp) peptide was reported. ES and the peptide were responsible for an accumulation of the Et–peptide–Taxol hydrogel in the breast tumor, and sustained release of the drug inside the cells.^87^

The cancer-associated fibroblast (CAF) as a kind of stromal cells can enhance the growth and invasion of cancer cells, and also promote metastasis and tumor growth.^88^ Hence, targeting CAF can be a right choice for cancer immunotherapy.^89^ To stop the activity of CAFs, a prolonged, sustained, and targeted drug exposure is necessary. In a study by Yaping Li,^90^ an injectable peptide hydrogel was designed to regulate CAFs and improve chemotherapy. Other vehicles (*e.g.*, nanoplatforms, such as lipid-coated calcium phosphate) have been utilized for CAFs targeting. For instance, by loading lipid-coated calcium phosphate with hydrophobic drugs like quercetin or genes, promoting the targeted accumulation and normalizing immunosuppressive TME can be enhanced. Leaf Huang *et al.*^89^ reported an excellent review on DDSs targeting CAFs. Cellulose-based gels are suitable for biomedical application. This is due to it being a renewable and biodegradable polymer that is a highly hydrated porous soft material with supreme mechanical properties. For instance, the encapsulation of drugs within nano-cellulose hydrogels results in the targeted release.

Genes containing small interfering RNA (siRNA), DNA, and micro RNA (miRNA) are known as an intermediary for CAFs modulation. Researchers are motivated to design a suitable delivery vehicle for having a sustained gene release in the target site. Regarding this, an injectable hydrogel, as an anti-cancer agent, was engineered to provide a sustained gene release (for 16 days) and overcame multidrug resistance.^91^

Collagen, chitosan, silk fibroin, albumin, hyaluronic acid (HA), and gelatin are widely employed for DDS. These biomaterials can be used in different forms, including hydrogels. Scientists have worked on tumor-targeted drug delivery systems employing HA.^92^ Not only HA, but also its derivatives have numerous applications in medicine due to their excellent physicochemical and biological characteristics. Moreover, it has the ability to bind to the specific receptors^93^ and result in targeted drug delivery.^94^ HA is a substantial carrier for antitumor-drug delivery, and also increases the residence of the drug in the targeted site. Remarkable results have been achieved, but there are still uncovered issues in the studies. For example, various types of synthetic methods for HA-drug conjugates affected the clinical uses.^92^ An injectable DDS for sustained cancer treatment was designed by Xin Chen^95^*via* the fabrication of a hydrogel (azobenzene and α-cyclodextrin-functionalized hyaluronic acid) and AuNBs (gold nano-bipyramids)-conjugated MSNs (mesoporous silica nanoparticles). This design was capable of introducing a microenvironment with a precious loading of anticancer drugs in and around the tumor tissue for periods, and suppresses the resumption of the disease. As mentioned previously, chemotherapeutic drug delivery can boost the treatment of the primary tumors before or after surgery. Recently, scientists synthesized a DOX-loaded Hydrogel by combining nanostructured lipid carriers and bacterial cellulose hydrogel, which depicted a considerable decrease of the tumor size, local drug toxicities, and also metastasis occurrence.^96^

In another research study, CMC-DOX polymer-drug hydrogels were synthesized to study the malignant melanoma form of skin cancer. Therefore, a platform was invented based on colloidal polysaccharide-drug nano complexes, generating anticancer hydrogels, which illustrated the new horizons for skin cancer therapy that utilizes transdermal drug delivery CT.^97^

Self-healable hydrogels are another promising type of hydrogels for cancer therapy. In the case of advanced staged tumors that cannot be removed by surgery, embolization is an option to gradually remove cancer cells due to the cells not receiving blood.^98^ Different biomaterials, including hydrogels, are recruited for embolic and chemo-embolic applications.^99^ To block blood flow towards the tumor site, a three-component self-healable hydrogel is designed to assist in the rapidly targeted embolization.^100^

#### Sensitivity

Many factors affect the rate of DR. There are various types of hydrogel-based DDS, including pH-sensitive, photosensitive and thermosensitive hydrogel (Table 2). Also, DDS can be sensitive to the salt concentration, electric potential, ionic strength, ultrasonic, light, electric-magnetic field, electric current and biomolecules for local cancer therapy.^101,102^ Among various stimuli, pH and temperature are the primary choices, as they are simple for understanding^103^ any disparities in the body's temperature or pH, which may result in distinct immune responses.^104^

Acid-base balance (pH), which is done by several mechanisms (*e.g.*, kidney and respiratory functions), is necessary for cell survival. Because of the intensive lactic acid production and respiratory carbon dioxide, cancer cells are subjected to large acid-base fluxes. Cancer cells can grow in an acidic environment, but they are unable to survive in alkaline conditions (high pH).^105^ Besides, the pH in the extracellular matrix (ECM) of solid tumors is mostly lower than that in healthy tissues because the glycolysis reaction in cancer cells has a higher rate.^106^

Providing a less cancer-friendly environment (low pH) will help scientists in fighting against cancer. So, pH-sensitive hydrogels can be designed based on those polymers, in which their ionizable chemical groups donate or accept protons.^106,116^ For instance, a multifunctional pH-responsive polysaccharide-based hydrogel can be invented as a new carrier of the drug (DOX). This new hydrogel is biocompatible and biodegradable, and also can be introduced as CDDS.^117,118^ In 2018, estrone (ES)-modified pH-sensitive glycol chitosan nanoparticles (GCNP-ES) were studied by Chunhua Yin *et al.*,^119^ focusing on drug delivery in breast cancer. Notably, GCNP-ES revealed the pH-responsive separability characteristics, while remaining stable under long-term storage and lyophilization. In another study, an injectable and pH-sensitive hydrogel (DF-PEG-PAHy/BPNSs) was engineered for combined chemo-photothermal cancer treatment. The DR of PTX-loaded GCNP-ES (PTX/GNCP-ES) was lengthened with noticeable pH sensitivity.^119^ PTX/GCNP-ES revealed greater agglomeration at the tumor site, which caused more tumor inhibition than PTX/GCNP and PTX solution. The similar results have been reported based on the *in vitro* cytotoxicity experiments. The DOX-loaded micelles revealed a greater prevention of the migration and proliferation of HeLa cells than free DOX by decreasing the pH from 7.4 to 5.0.^120–122^ In a study done by Wu *et al.*,^122^ the release profiles at pH 7.38, 6.67, 6.17 and 5.03 exhibited a rapid release at higher pH. For the lower pH values in another study^123^ using poly(NIPAM-*co*-acrylic acid) nano-gels, a quick release of the drug (DOX) was observed at lower pH. We believe that this opposite reaction is one of the main challenges to reach a unique strategy.

Cancer (tumor) cells have higher temperatures because they are continuously replicating and proliferating, and this process releases more energy.^124^ Therefore, the temperature of the tumor environment can be considered as a factor to detect changes of the tumor area. Hence, evidence showed that researchers designed thermo-sensitive hydrogels as a therapeutic carrier to reach a controlled release in clinical research, including tumor removal.^125^ For example, a DOX-loaded thermo-responsive self-healable hydrogel has been engineered by Wenjuan Li.^126^ It was demonstrated that the DOX was released faster at 40 °C (nearly 50 percent of the medicine within two h) compared to that at 30 °C (almost 25 percent of the medicine within two h). Since the temperature of the tumor is higher than that for healthy tissues, there is a remedy to prevent or remove the tumor by implanting a kind of hydrogel like that one. Effective therapeutic methods of melanomas are still limited. So, for finding a superior anti-tumor efficacy, a thermosensitive hydrogel based on poly(γ-ethyl-l-glutamate)-poly(ethylene glycol)-poly(γ-ethyl-l-glutamate) (PELG-PEG-PELG) has been developed to study the chemo-immunotherapy system of IL-2, DOX, and IFN-γ for the treatment of melanoma by testing the invented hydrogel on B16F10 cells *in vitro*. Results revealed a noticeable performance against the B16F10 melanoma xenograft with lower systemic toxicity.^127^

Also, dual pH and thermo-responsive DDS has been interesting as the next-generation therapeutic medicines.^128,129^ Scientists could successfully synthesize dual sensitive and responsive hydrogels encapsulated with proteins^130^ and 5-aminosalicylic acid^131^ using different types of polymers through *in vitro* studies. In another research study, a thermosensitive hydrogel (hyaluronic acid-chitosan-*g*-poly (*N*-isopropyl acrylamide)) for the intra-tumoral delivery of DOX to the breast tumor has been engineered successfully.^132^ The chitosan/hyaluronic acid/β-sodium glycerol phosphate-based hydrogel loaded with DOX was another dual sensitive structure, which is adhesive to the cancer cell (Hela). It was also injectable, and turns into hydrogels when the temperature is raised to that of the body.^133^

Furthermore, by controlling the conditions in hydrogel processing, DR can be controlled as well.^134^ The rate of DR can be regulated by the polymer degradation rate, redox conditions, the physical state of the nucleus, the position of the drugs in micelles, the length of the micellar nucleus fragments, the size of the drug molecule, and the drug-loading rate.^120^ Apart from the process, the selection of the material is also significant. It depends on many factors, such as the size of the hydrogel, inherent properties of the drug, surface characteristics, including electrical charge, permeability, biodegradability, and importantly, the route of the drug delivery.^135^

It has been shown that the binding of the drugs to the polymer core (hydrophobic and hydrolytic cores) and the variation of the methyl dioxanone units are known as other factors that control DR, which led to the sustained release profile over 5 days.^136^ Also, altering the concentrations of aqueous polymers, like poly(ethylene glycol)-poly(amino carbonate urethane) in hydrogels, could cause sustained or controlled DR.^137^

According to the particle size, hydrogels can be divided into microgels and nano-gels.^138^ Encapsulated microgels containing drugs and therapeutic agents, such as anti-cancer compounds and proteins, could be successfully prepared. Microgels have similar polymeric chemical properties and different physical properties, including viscosity, surface area, and thermal response with hydrogels.^139,140^ Nanogels, known as the swollen amphiphilic/hydrophilic aqueous dispersions of hydrogels, are a promising carrier system for DR. Nanogels made of poly(methyl acrylic acid) and chitosan revealed pH-responsive properties with controlled DR.^122^ Moreover, *via* chemical modification, they could be employed to combine different ligands for targeted drug delivery. Hyperthermia therapy, a process that leads to high temperature in the tumor site (40–45 °C), is almost used with other current cancer treatments, including radiotherapy and chemotherapy. Hyperthermia may make cancerous cells more sensitive to radiation, but it can cause damage to the other surrounding tissues. Hydrogels proved to have excellent potential to control the local heating. Hydrogels can be engineered as a multi-therapeutic system. They can be pH or thermo-sensitive while loaded with various therapeutic agents and provide local heating.^141^ For instance, a thermosensitive nanocapsule hydrogel loaded with iron oxide nanoparticles was employed to evaluate magnetic hyperthermia therapy as a cancer treatment. The results of this research revealed a remarkable *in vivo* anti-cancer effect after 4 cycles of magnetic hyperthermia therapy. It was also reported that the surrounding healthy tissues remained undamaged. Overall, hydrogel-based delivery systems showed their clinical use potential for cancer therapy by having control over the delivery and release of different therapeutic agents, including macromolecular and small-molecule drugs.

### Hydrogels for cancer 3D models

To supersede employing conventional treatments, including radiation, chemotherapy, and surgery, scientists have made many attempts to study and discover antitumor drugs. Clinical trials not only are too costly, but also often result in numerous failures. These mainly are because of the lack of suitable preclinical models to study. Cancer cells in a solid tumor are kept strictly with ECM as a non-cellular physical and functional support for cell survival, tissue integrity, and expansion. ECM hydrogels are derived from many tissue sources and employed to study numerous disease models. ECM comprises mostly elastin, collagen, laminin, fibronectin, and proteoglycan.^142^

TME is a heterogeneous system that involves the ECM, cell–cell interactions, and environmental stimulations, and is one of the significant factors to determine the tumor fate. 2D Cell culturing, as an *in vitro* model, relies on adherence to a flat surface to supply mechanical support for the cells. This model is employed to study cellular responses to stimulations from biochemical and biophysical cues. In 2D monolayers, cells can access a similar amount of growth factors and nutrients of the medium, which leads to homogenous growth and proliferation.^146^ Although 2D cell cultures are employed widely in drug discovery and development research, the generated data from them often do not predict what might occur *in vivo*,^147^ and does not simulate the real microenvironment of cells. Thus, making an artificial environment that mimics the behavior of the actual tissues can be useful.^143^ The use of 3D scaffolds as cancer models not only will affect the drug screening,^144^ but also stops or lower the number of animal testing carried out in preclinical studies.^145–147^ The application of the 3D cell culture systems gives more feasibility to control the shape, porosity, chemical composition, structure and stiffness of the 3D matrix. This influences the proliferation, migratory capabilities, and mimicks the cell–cell interactions and normal cell-matrix. For instance, recent progress in biomedical and tissue engineering improves 3D models by considering the tumor microenvironment, which is vital for metastatic progression and vascularization.^151^

These 3D scaffolds can be formed by materials that resemble the ECM components. There are various materials, including the natural sources like collagen and hyaluronic acid (HA), and the synthesized materials such as poly(lactic acid) (PLA), poly(ethylene glycol) (PEG), and others. The evidence shows that two common 3D cell culture systems are becoming more popular among scientists: tumor spheroids (TS) and scaffold-based models.^148^ In this section, we provide a short review of these models, which are mostly employed for clinical research.

CAFs are complicated cell types within the TME, and have been demonstrated to be a vital component in helping cancer progress.^149^ On the other hand, TRACER (The *tissue roll* for *analysis of cellular environment and response*) is a novel approach that helps to analyze cellular behavior, and phenotype in hypoxic gradients and distinct cell populations from particular microenvironments.^150^ In a research study focused on patients suffering from neck and head cancer, Alison P. McGuigan *et al.*^151^ integrated derived CAFs into the TRACER platform to establish a greatly hydrogel-based tunable 3D co-culture tumor model that mimics the CAF-TME interactions in head and neck tumors, and allows for the patterning of different cellular compartments and cell separation from each region for phenotypic assessment. According to the reported data, the introduced 3D co-culture system is a highly versatile model that can be employed to provide a more exceptional understanding of the cancer-stroma interactions, and their influence on the cancer cell reaction to the therapy.

**Fig. 3:**
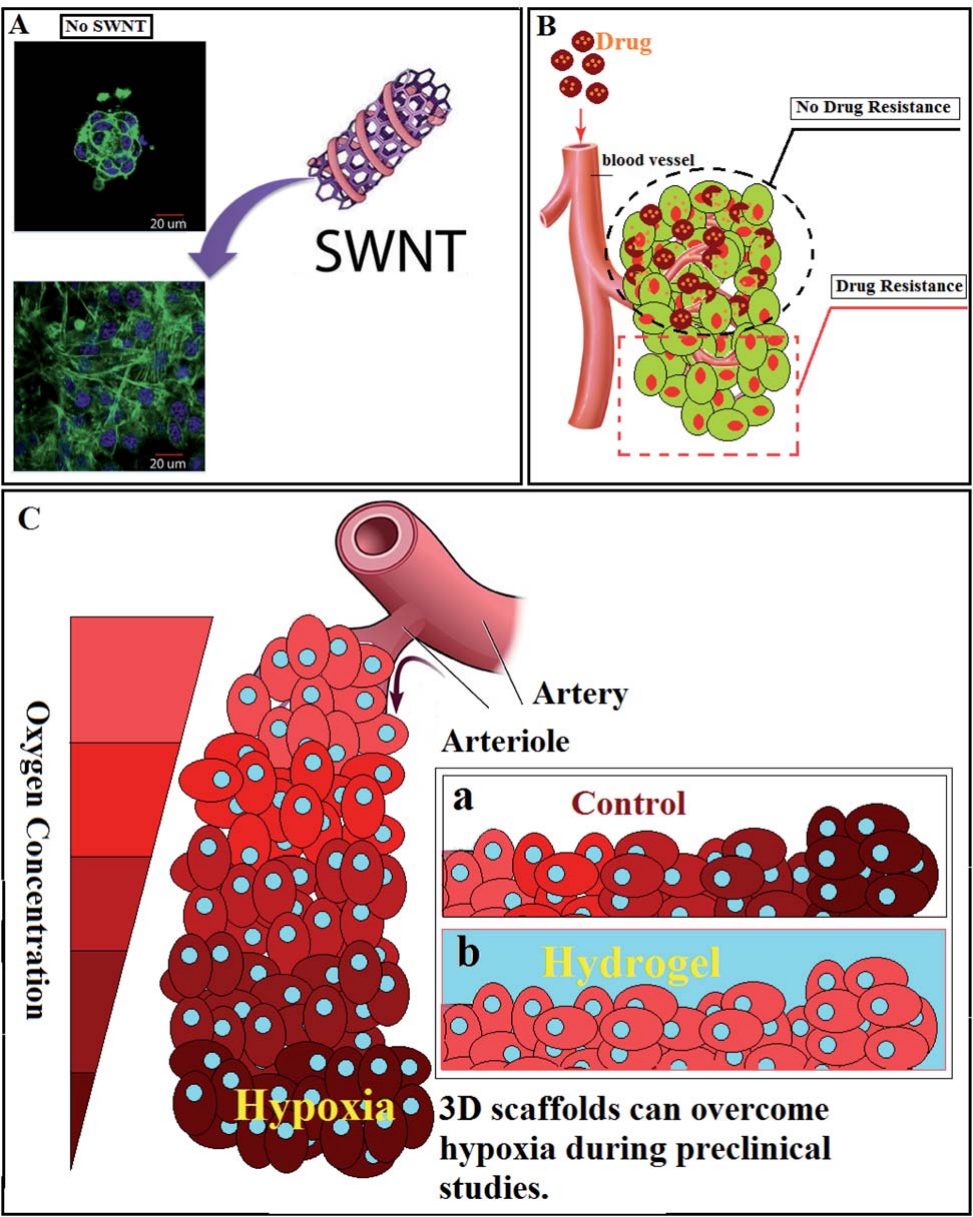
(A) Effect of the presence of SWNTs in the peptide hydrogel on cell behavior.^152^ (B) Tumor resistance against drugs. (C) Hypoxic cancer cell: (a) lack of oxygen in tumor site, (b) hydrogels as cell culture media can provide enough oxygen to the tumor site.

In another study, the usage of hybrid self-assembling peptide (EFK8)-carbon nanotube (SWNT) hydrogels for TE and *in vitro* 3D cancer spheroid formation was reported.^152^ Comparing the engineered hybrid hydrogels with EFK8-only hydrogels revealed an increase in the attachment, spreading, proliferation, and movement of NIH-3T3 cells (Fig. 3A). Cells could proliferate rapidly on the hydrogel containing SWNT. It was confirmed that not only in 2D but also in 3D cultures, cells could spread uniformly and have more movement on the hybrid hydrogel than on an EFK8 hydrogel. Moreover, it revealed that increasing the peptide concentration resulted in raising the compressive modulus of the resulting hydrogels. Finally, it was demonstrated that incorporating SWNTs into the peptide hydrogels will give more applications to these hydrogels in TE, and can expand our insights into the cell-biomaterial interactions.

Restricted penetration of therapeutic agents in solid tumors is a potential cause of drug resistance (Fig. 3B). According to the previous studies, cellular packing density and cancer cell adhesion alter drug penetration.^153^ Apoptosis, cytotoxicity, and infiltration of conventional drugs can be studied with the aid of hydrogels. In one study, an alginate-based 3D hydrogel has been designed as a matrix to mimic TME, duplicating the physiological conditions, and study the effect of various anticancer agents and disparate molecular pathways. The highly specialized TME is usually named as the cancer stem cell (CSC) niche. The engineered matrix revealed that CSC niches could be studied more appropriately due to the intrinsic 3D nature of CSCs.^154^

Hypoxia-Inducible Factor-1 (HIF-1), is one of the prominent features of advanced cancers (Fig. 3C).^155^ Hence, hypoxic O_2_ gradients have captured the attention of scientists. In one study, researchers could engineer a hydrogel to assess the effect of hypoxia on sarcoma cell invasion and migration. Through this research, O_2_-controllable hydrogels were introduced as a prognoses system to evaluate the primary states of the metastatic process.^156^ The 3D collagen scaffold revealed a higher reactive oxygen species (ROS) level, and showed more resistant to cisplatin in comparison with the 2D model. The 3D culturing cells demonstrated an increased level of ROS, representing a physiological variation or the establishment of a microenvironment that simulated tumor cells *in vivo*.^157^

Breast cancer as a common cancer among women has been one of the important topics in preclinical studies. Engineering a 3D model of breast cancer can provide essential observations and data, which may accelerate research studies and precise outcomes. For example, a newfound engineered 3D TE hybrid hydrogel system made of collagen and alginate has been introduced.^158^ Through this hydrogel, physical analysis and biochemical signals that impact cancer progression and also drug screening were studied in detail.

Cell migration to the surrounding tissues is the primary requirement for metastasis. On the other hand, adipocyte cells (as one of the significant stromal cells in the human breast) play a significant role in gland development and breast tumorgenesis.^159^ In a research study, the *in vitro* breast cancer cell migration and the influence of adipocyte-mediated on it have been studied by employing an engineered hydrogel-based human adipose/collagen model.^160^ Within this study, an adipose-based model revealed more cell migration over empty scaffold controls, and so it has been suggested that this 3D *in vitro* model can be employed as an assay to analyze the cancer therapeutic effect in individual medicine strategies. The making of an interpenetrating network hydrogel system from collagen and alginate, titled CoAl-IPN, to study cancerous breast tissue stromal niche was reported by Cao *et al.*, and was proven to be reliable as a versatile platform for cancer progression.^161^ Furthermore, in the topography and mechanical property points of view, the HA hydrogel formed *via* hydrazone-/photo-dual crosslinking process demonstrated more similarity to a real breast tumor tissue, and HA was also able to promote cell migration and *in vivo* tumorigenesis.^162^

Similarly, more 3D hydrogel-based environments have been engineered for other types of cancer. For instance, 3D hydrogel-based models with the purpose to recapitulate, regarding ovarian cancer, have been developed.^163^ Furthermore, an *in vitro* model has been introduced for lung cancer to study metastasis and recapitulate cell invasion, which when compared to 2D models, showed more biomimetic behavior.^164^ The 3D alginate hydrogels, as one of the prevalent *in vitro* environments, have been employed for *in vitro* metabolic, radiobiological studies of cancer cells and drug screening. Within this research, it was demonstrated that hydrogels are a great versatile candidate for extracellular flux analysis, modeling metabolic changes, hypoxia and hyperpolarized magnetic resonance spectroscopy.^165^

Many efforts were made to employ the 3D hydrogels for fundamental studies on the cell-matrix or cell–cell interactions in pancreatic ductal adenocarcinoma (PDAC). Researchers could use hydrogels to recapitulate the PDAC tumor matrix by driving the HA-based hydrogels, collagen gel, and Matrigel from TME, or inspired by components in the TME to produce pathophysiologically related circumstances, compositions, and contexts to support the PDAC cell fate processes.^166^

The most common primary malignant tumor of the bone is called Osteosarcoma (OS). Gene therapy, as a novel method, can be employed for OS treatment.^167^ Although no 3D model is capable of fully reproducing the heterogeneity and complexity of OS by considering the TME, the 3D approach could simulate the micro-environmental physiology of tumors. The report of the analyzed methods revealed that all 3D OS culture methods, including scaffold-free or with a scaffold, represent a beneficial tool to recapitulate the complex TME and spheroid known as the most suitable system.^168^

A 3D endothelialized vesicle equivalent as a bladder collagen-based cancer model was developed.^42^ It can be used as a trustworthy model for drug response appraisal, mechanical analysis, and identifying targetable molecules that potentially decrease animal experiments and help in the toxicological evaluation of anti-cancer medicines.

To recapitulate the cancer stages and human tumors, providing a 3D environment that mimics the ECM is necessary. Owing to the 3D hydrogels, the *in vitro* study of various tumor cells, modeling tumor angiogenesis will be easier. Furthermore, changing the conditions and main parameters, and even drug screening will be easier and provide more reliable conclusions. By combining this technology with current techniques, like microfluidic devices, an advanced micro-vessel model can be created to reach more accurate new results.^169^

### Hydrogels for cancer-model 3D printing

Common strategies used for the fabrication of an *in vitro* cancer 3D model involve cell seeding, microsphere, encapsulation, microfluidic system, 3D printing (3DP), and 3D bioprinting (3DBP). Studies of cancer development, and the subsequent development of cancer therapies, are very challenging due to the complex interaction of the biological factors that cause disease progression. To study cancer cell behavior, including their physical functioning and migration, an *in vitro* 3D hydrogel environment was created using 3DP. 3DP as a computer-assisted method is another new technology to engineer the TME. This is because it allows for having a multi-level spatial control of the cell organization and assignment of biomolecules/materials. 3DP is a potent solution to overcome the obstacle of TE, including the construction of a complex biological architecture, high precision-shape of the tissue structures, and cell differentiation/proliferation.

3DP suggests an entirely new method to make a complex tissue comprising complex 3D microarchitectures, like cartilage, skin, bone, and blood vessels. This technology also simplifies the delivery of different cell types and polymers into a single construct through the simultaneous incorporation of micro-channels that help the diffusion of nutrients and oxygen. Moreover, 3DP models not only provide a remarkable study of cell–cell communication and cell-matrix interactions, but also can promote and facilitate drug testing and cancer therapy development.^170^

**Fig. 4:**
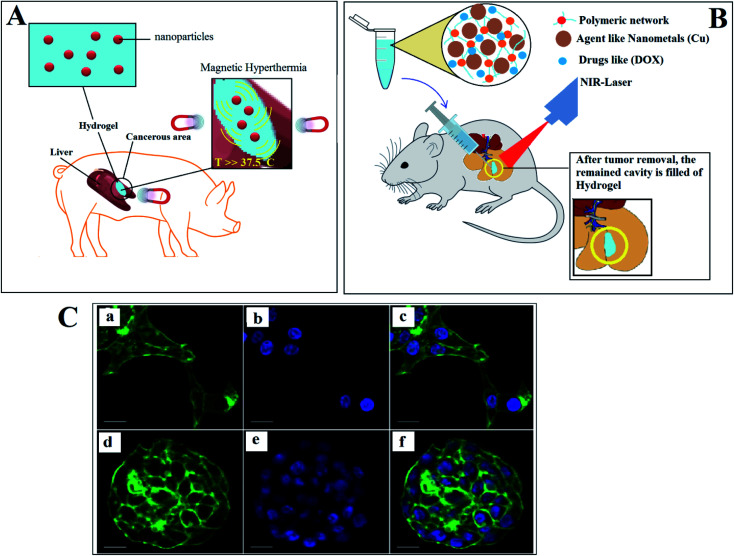
(A) Employing hydrogels loaded with nanoparticles for boosting magnetic hyperthermia. (B) Scaffold/hydrogel having practical photothermal effects. (C) Confocal microscopy of HCC70 cells cultured in monolayer and hydrogel environments. (a_c) In monolayer culture, (d_f) In hydrogel environment.^178^

Magnetic hyperthermia is one of the novel methods for tumor elimination, which by generating heat and applying magnetic fields, the cancerous tissues are removed (Fig. 4A). The efficacy of this method depends on the degree and pattern of the generated heat, and also the diffusion and distribution pattern of the magnetic nanoparticles. The *in vitro* models are good candidates to investigate and monitor the diffused magnetic nanoparticles, generated heat, and changes in tumors.^171^ Using 3DP revealed its ability to simulate the most essential characteristics of the cancerous tissues, such as shape, vascular network, and others.^172^ The bone invasion of oral cancer has been studied by introducing a 3D printed tissue-engineered model. Compared to animals or 2D models, it was a more reliable human cell-based alternative, unlike *in vitro* applications due to: (i) providing a representative tool to research and engineer oral cancer at various anatomical levels, and (ii) to be used to evaluate diagnostic or therapeutic approaches to manage Oral Squamous Cell Carcinoma (OSCC) as the most common oral malignancy in the future.^173^

3DP makes 3D spheroids able to accurately mimic some features, like physiological responses, drug resistance mechanisms, spatial architecture, secretion of soluble mediators, and gene expression patterns of solid tumors. Vitor M. Correlo *et al.*^174^ studied TS technologies, which emerged for 3D *in vitro* cancer modeling. TS are a suitable and robust representative for the 3D organoid-like framework. TS comprise the intercellular signaling of cancer. It not only can be utilized as a drug screening platform, but also provide more accurate data.^175^

In the case of using 3DP to fabricate medical structures and scaffolds for distinct medical fields, it is essential to select suitable materials. Gelatin, alginate, chitosan, fibrin, agarose, poly(hydroxyethyl methacrylate), poly(vinyl alcohol), poly(ethylene glycol), and combinations of them are some of the hydrogels used in 3DP. As mentioned previously, gelatin and alginate, known as two of the most common biomaterials due to their bio-mimicry, biocompatibility and mechanical properties, are used in 3DP. The combination of alginate and gelatin results in characteristics similar to that of native tumor stroma.^176^ A 3D model, which mimics the *in vivo* microenvironment, was also tested successfully by embedding breast cancer cells and fibroblasts in the hydrogel and in a long-term cell culture (>30 days).^176^ The migration, proliferation, and interaction of the CAF cells with the multi-cellular tumor spheroids were observed in this model. By using this 3D model as the co-culture systems in studies, the dependence of tumorigenesis on the stroma composition can be investigated.

Breast tissue loss and the high local recurrence of cancer are the main threats after surgery. Filling the cavity with therapeutic-agent hydrogels is a promising approach to prevent cancer relapsing. Photothermal therapy uses electromagnetic radiation, often in infrared wavelengths, to treat different medical conditions, including cancer (Fig. 4B). To apply photothermal treatment, it is crucial to synthesize a scaffold/hydrogel having practical photothermal effects. In one study, a porous scaffold (dopamine-modified alginate and PDA) was fabricated using 3DP to overcome the local recurrence of breast cancer. The results demonstrated the potential of 3DP in the fabrication of a hydrogel with mechanical characteristics that matched those of breast tissues, and its impressive photothermal effects.^177^ Methods for the ultrastructural analysis of Hepatocellular Carcinoma 70 (HCC70) cells cultivated in a monolayer culture and 3D hydrogel conditions were developed by Elba E. Serrano *et al.*^178^ The protocols to preserve the Triple-negative breast cancer (TNBC) cell line HCC70 in a monolayer culture (2D) were established in a membrane matrix hydrogel, which is commercially available. In a monolayer culture, the shaped flattened cells spread disorderly (Fig. 4A–C), while the cells maintained in a 3D hydrogel ended in multi-layered spheroids (Fig. 4D–F). The results showed the benefits of this method to study and analyze the transmission electron microscopy (TEM) images of the hydrogel cultures of both cancer and healthy cell lines.

**Fig. 5:**
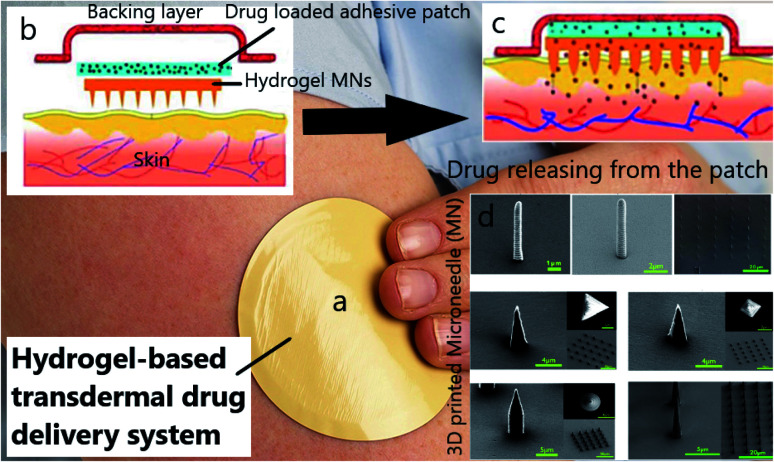
(a) TDDS patch, (b) structure of a TDD patch, (c) drug diffusion within the skin's capillary system, (d) SEM images of 3D printed microneedles (MN) with different shapes.^18^

Apart from the simulation of tissues, drug delivery for cancer therapy is known as another aspect of 3DP technology. Patches are one of the most exciting vehicles for the transdermal drug delivery system (TDDs) due to a unique design (Fig. 5a) and a painless injection. Patches are designed as a ‘reservoir patch’ and ‘matrix patch’. To engineer a patch, a drug-containing hydrogel is first made, and then an adhesive substance is added to the polymer to prevent patch separation from the skin. By applying the patch on the skin, drugs begin to diffuse into the skin through the needles (Fig. 5b, c). Patches contain specific microneedles with various architectures (Fig. 5d). Microneedles can be fabricated in multiple shapes and heights (25–2000 μm), which assure that the drugs will be released into the depth of the skin's capillary system. In spite of the progress of patches for clinical trials, there are some weaknesses that must be improved. For example, it takes a long time for the drugs to reach the targeted tissue through the skin. In addition, not all loaded drugs are expected to reach the target site. This is because there is a possibility of drug crystallization through the skin or even in the patch. Many publications^18,179–181^ studying the various applications of 3DP centered on TDDs. Regarding breast cancer, an engineered transdermal patch showed an impressive release of two mg anastrozole, with damage to the healthy surrounding tissues.^182^ In another study, a HA-hydroxyethyl cellulose-based hydrogel was investigated for transdermal isoliquiritigenin delivery. It was shown that the release kinetics of this hydrogel follows the Fickian diffusion mechanism, which guarantees its TDD application.^183^

**Fig. 6:**
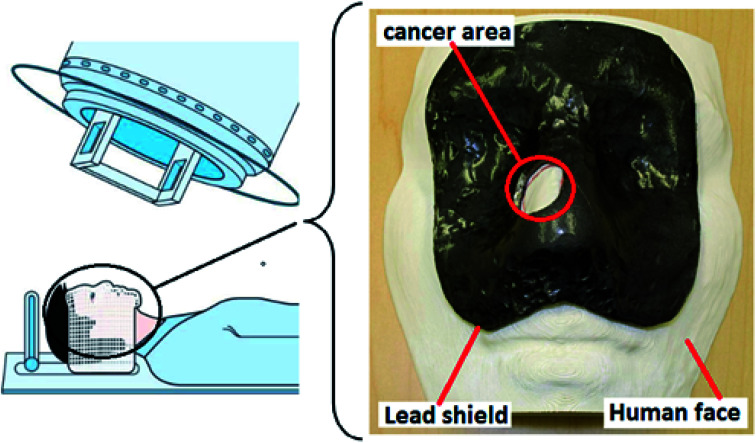
Using a 3D-printed lead shield during radiotherapy for facial skin cancer.^184^

To prevent damaging the healthy tissues around the cancerous area during RT for facial skin cancer, lead shields are employed. In this case, by having the topography of the patient's face, a lead shield can be 3D printed. Patients who used the lead shield were comfortable during RT (Fig. 6).^184^ Considering lung cancer, a 3D printed hydrogel was employed to improve patient comprehension for surgical resection (stage I). Through this research, 20 adult patients with lung cancer were randomly assigned to 3DP. The 3D printed hydrogel-based model was technically implementable, and capable of improving the patient comprehension for surgical resection.^185^ In another study, to fabricate an innovative 3D cell culture for lung cancer, 95D cells were co-cultured with the 3D printed agarose and alginate-based scaffold. Results revealed that cells could accumulate into the spheroids, and invaded, proliferated and migrated into the surrounding structure.^186^

Owing to the 3DP technology, doctors and medical engineers can create 3D models for different medical applications before the surgical procedure. Nasal skull base tumor is an issue studied by researchers of the Department of Otorhinolaryngology Head and Neck Surgery, and the Department of Neurosurgery of Tianjin Huanhu Hospital. In this study, they used 3DP technology, and illustrated the relationship amongst the tumors, skull, and adjacent blood vessels.^187^

The vertebral column, known as the backbone and spine, is the most prevalent and frequent site of bone metastasis. Remaining defects due to the surgical elimination of backbone metastases cannot spontaneously be healed, and bone grafting is required. Because systemic doses of chemotherapeutics (such as DOX, paclitaxel, or cisplatin) can cause side effects, a 3D printed scaffold was engineered as a bone substitute to aim the targeted chemotherapeutic delivery *in situ*. This study reported reduced side effects and accelerated bone repair.^188^ In another case, 3DP is used for guiding scientists on bone tumor resection, so that not only it can be personalized for patients and employed for bone tumor surgery, but it is also noninvasive, easy to use, and informative.^189^

A newly published paper revealed noticeable information about colon cancer cell migration by using 3DP technology. Within this research, hydrogel-based 3D scaffolds were 3D printed to study the functionalities of TME (*in vitro*) to introduce a new platform for novel therapeutic strategies. Thus, researchers could assess cancer cell migration and study tumor metastasis therapy using the engineered 3D printed models.^190^

Overall, hydrogels (with the help of 3DP) provide modern facilities for researchers to augment their studies in a more accurate and cost-effective way. Scientists could make a 3D-printed mandible template (after cancer) to accelerate product development, while lowering the manufacturing and clinical expenses.^191^ Moreover, 3DP enhances the likelihood of a personalized remedy or a unified treatment for each patient. In spite of the numerous studies in this area, there is still a quick need to develop and unite the proposed methods to aim for a complete strategy.

### Hydrogels for cancer-on-a-chip (COC)

Many drugs are synthesized and tested in laboratories for various illnesses. However, before distribution, they must pass preclinical trials and clinical translation, and also be approved by the Food and Drug Administration (FDA), which generally prolongs the process for several years.^192^ Scientists have been trying to find a way to shorten this time, and study the efficiency of drugs in as short a time as possible at a lower cost.

On the other hand, the way of studying different drugs has been challenging for scientists. As previously discussed, using various 2D cultures, animals and even 3D hydrogels cannot truly confirm the effectiveness of a drug because the culture medium or drug substrates are made of materials that are unable to accurately and adequately mimic the ECM. Thus, medications may result in side effects and be refused by the patient's body, and researchers are still trying to overcome this problem.

**Fig. 7:**
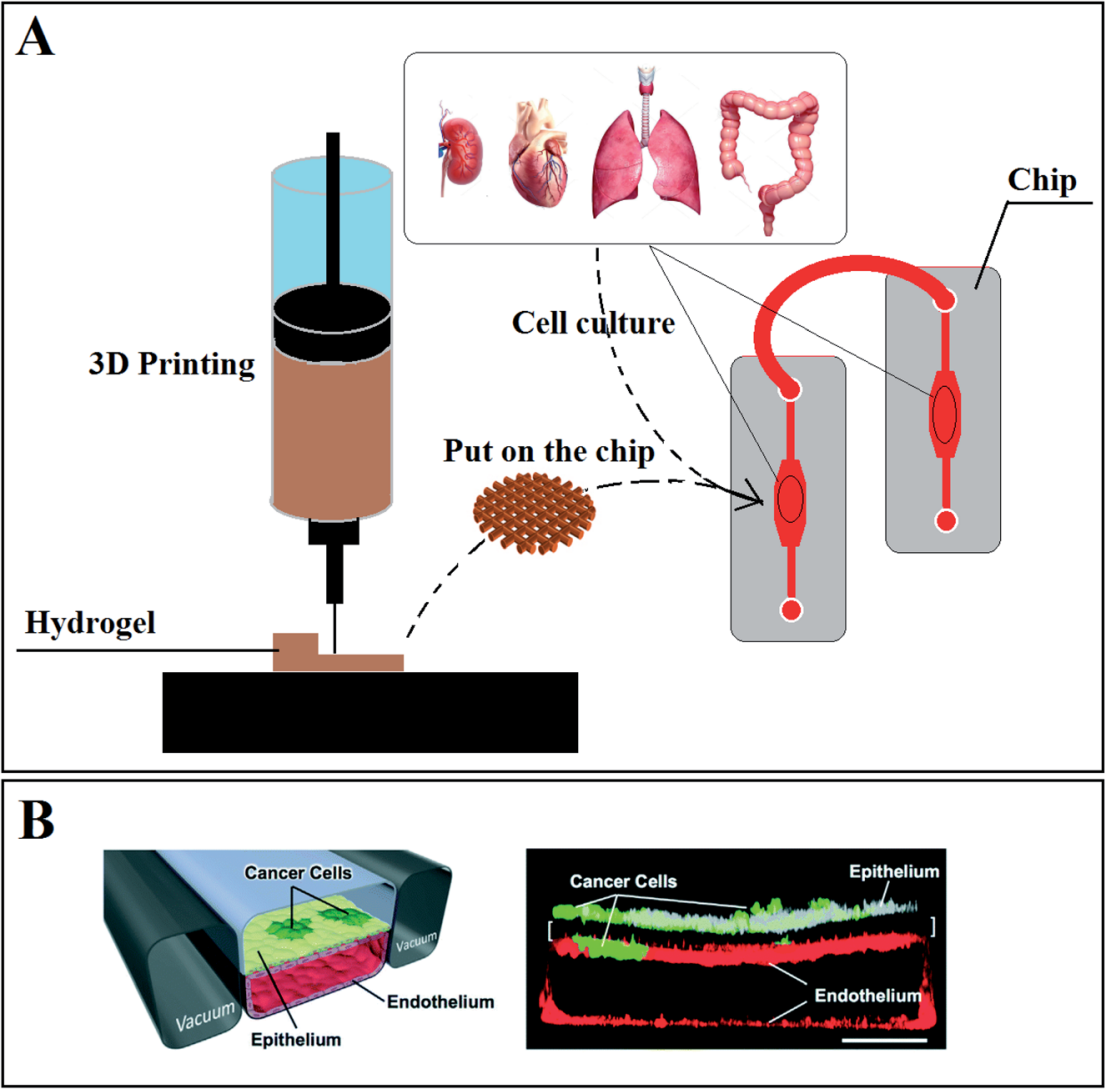
(A) Using 3D printing to make a suitable scaffold (hydrogel) to place in chips to monitor cell reaction and functionality in different conditions. (B) Schematic diagram of lung-on-a-chip: (A) a cross-section of the microfluidic chip with human lung epithelial tumor cells (cultured on the upper surface of a porous membrane). (B) confocal fluorescence micrograph of a cross-section of the assembled chip when cells were cultured for 7 days without breathing motions (scale bar, 200 μm).^207^

OOC, known as a microfluidic system, is a new technology that has been revealed lately to focus on different types of diseases,^193^ like the human intestine,^194^ inflammation,^195^ skin disease,^196^ liver disease,^197^ kidney disease,^198^ cancer^199,200^ and drug research^201,202^ and others. This technology helps researchers to decline and approximately stop using animals and other inhumane strategies for drug assessment. Designing the organ's functions on chips is a significant attempt to create similar environments in microscale devices named ‘chip’ to mimic the real organ.^22^ Generally, COC or OOC can be a combination of hydrogels, 3DM, and 3DP. First, the platform (scaffold) of the tissue or organ is made *via* 3DP or other fabrication methods, and then it is placed on the chip (Fig. 7A). So, a chip comprises three main parts: hydrogel (scaffold), microchannels, and the structure of the chip.^203^

Comparing OOC with other conventional technologies has shown that this powerful predictor and the tiny chips can shorten the period of the drug approval procedure, reduce the use of animals, and reduce the costs of the drug cytotoxicity analysis. The chip provides easier and more accurate monitoring of the cultured cells, and it also helps the cells to access the signals and nutrients uniformly by enabling the media to recirculate through the platform.

Cancer On a chip (COC) is assumed as the main idea to develop a new predictive cancer model with advanced capabilities of mimicking the ECM. Many papers have been published regarding the employment of COC for cancer studies and remarkable progress in improving COC for metastasis research studies. Multiple OOC can be connected to recreate more complex cell–cell interactions.^204^ Drugs can affect the target sites at specific rates in a well-controlled manner by OOC *via* implantation, integration, automation, localization, and the accurate control of OOC parameters.^205^ In the following section, we present a summary considering the recent and related works on COC models.

As previously mentioned, hypoxia is known as a problem in standard cancer lab models like 2D cultures. Cancerous tissues were studied by Louise Orcheston-Findlay *et al.* because of the high proliferation rates of the cells.^206^ This study investigated the nano-scale properties with the help of hydrogels and a 2D culture in various oxygen concentrations. The proposed system can be used to study more anti-cancer drugs, and to evaluate the combination of hypoxia-activated drugs and different therapies. Signs of morphological altered cells in response to the O_2_ gradient have been detected, and TE areas (like stem cell differentiation) can benefit from controlling the O_2_ level *via* COC technology.

It has been shown that COC technology can be used to make *in vitro* human orthotopic models of lung cancer.^207^Fig. 7B shows a cross-section through the 2-channel COC equipped with a hydrogel-based 3D platform containing human lung epithelial cells. As can be seen, cancer cells can grow and proliferate, as well as in a real human lung. Proposed drugs could be tested to check drug responses by using this chip as a real lung model.

COCs provide easy manipulation of the liquid in the volume of microliters and dynamic control of the tumor-stroma communication,^208,209^ as well as tumor-ECM interaction.^210^ Moreover, they were restricted from more studies considering drug screening. This was because the various sized multicellular tumor spheroids existing on the platform might result in variable drug responses.^31,211^

To evaluate nanoparticle-based DDSs, an *in vitro* breast tumor model-on-a-chip has been developed by Dan Gao *et al.*^31^ to rapidly evaluate the DDS compared to a carbon dots (CDs)-based DDS dynamic transport behavior. The *in situ* cytotoxicity evaluation in one system can be evaluated by this microfluidic platform, which provides a correct and low-cost *in vitro* model to have a rapid and accurate drug screening in preclinical drug evaluation. Metastasis-on-a-chip has been studied as a form of COC to assess and evaluate the metastatic preference of cancer cells. Several microfluidic chambers were connected as the representatives of the liver, lung, and endothelial constructs under media fluid flow recirculation. This resulted in a better understanding of the mechanisms underlying metastasis.^212^ Similar works have been introduced as new and powerful platforms for cancer progression and metastasis.^213,214^ For instance, based on our report in previous sections and due to the imperfect mimics of cancer metastasis physiology by animal and 2D models, metastasis-on-a-chip was developed to model *in vitro* tumor progression, as a screen for drugs, and to characterize human metastasis. Through this research, there was the possibility of tracing the fluorescent colon cancer cells, which migrate from a 3D hydrogel-based gut to downstream liver constructs within a circulatory fluidic device system that reacts towards environmental manipulation and drug treatment.^215^ Blood-based liquid biopsies supply opportunities to detect early-stage cancer and anticipate cancer progression, monitor responses to chemotherapeutic drugs accurately, and personalized treatment.^216^ To study breast cancer, a 3D breast-cancer-on-chip, as a model containing the endothelial monolayer and ECM, was developed by Dan Gao and his coworkers to evaluate the dynamic transport behavior and *in situ* cytotoxicity of the employed hydrogel (carbon dots-polyethylene glycol-folic acid) as the platform. Consequently, the proposed chip was approved as a low-cost *in vitro* model for the rapid and accurate drug screening in experimental and pre-clinical studies.^217^

Tumor growth and metastasis are firmly relevant to angiogenic vascular networks. The integration between hydrogels, as a similar environment to TME, and COC help scientists to reach a fast and accurate characterization of angiogenesis phenomena through cell migration and interactions, and during cancer progression. One study revealed remarkable information about combining hydrogel-based microfluidic devices as COC. These new platforms could help scientists have more control over the spatial architecture, shear stress, complexity of the angiogenesis mechanism, and nutrient and chemical transport properties.^218^

Toxicity was investigated by introducing a liver tumor-on-a-chip equipped with a decellularized liver matrix-gelatin methacryloyl-based model by Shu Qi Wang *et al.* They demonstrated that this new chip can provide a better mimicking of the *in vivo* TME, and gives great promise for pharmacological and pathological studies.^219^ Owing to the COC, there is a likelihood of creating an *in vitro* model for lung cancer that can recapitulate tumor dormancy and cancer growth.^220^ Bone metastasis occurs in nearly 70% of those who suffer metastatic breast cancer. Researchers could engineer a bone-on-a-chip comprising a 3D collagen-based platform to assess cancer cells-host bone marrow cell interactions and also related physiological alternation.^221^

COC, as a new approach in science, integrates the hydrogels and chip to provide a rapid and efficient drug testing, model cancer metastasis, and also offers personalized cancer models. This combination reveals the details of the signals, chemical and cellular factor transportation in tumor progression and angiogenesis. The expansion of COC helps discover more information about the complexity of the microenvironment, the complex cell–cell, and cell-ECM interactions their interaction with other tissues, and drug resistance. However, before the on-chip platforms can be widely adopted for real medical application, various challenges are still uncovered and need to be addressed.

## Conclusions and future perspectives

Many patients suffer from cancer disease. Surgery following chemotherapy and radiation is the standard treatment, but hydrogels are known as a new facility for cancer therapy.^39^ In this study, hydrogels were introduced as the promising biomaterials in cancer clinical researches. We demonstrated that recent technologies, including 3D models, DDS, 3DP, and OOC benefit hydrogels to make progress in cancer therapies.

Culturing distinct cell types, considering both cancer and healthy cells in an environment, having high efficiency in mimicking ECM, is known as an extraordinary achievement. Hydrogels help study and monitor the interaction of cells towards changes in the environment, such as drugs, tensions, strength, nutrients, oxygen, temperature, pH, and others.^222^ Different parameters, such as cell migration, angiogenesis, metastasis, cell–cell, and cell-ECM interactions oxygen level, CAF, metastasis, hyperthermia, can be appraised by combining hydrogels with other approaches.^169,206,222^ The discovery of new more suitable hydrogels for cell culture is one of the main topics under survey. According to this review, many biomaterials are employed to study various sorts of cancers, but it must be considered that each hydrogel should be engineered properly to the primary tissue. Hydrogels are suitable candidates in CT and RT to lower the side effects by providing a targeted drug delivery.^63^ Moreover, hydrogels showed their potential as a culture media for different cancerous cells. In addition, the high ability of hydrogels in programming and the sensitivity to external stimuli have captured the attention of scientists.^43^ In spite of all these distinguished outcomes, there are still many challenges in cancer therapy that need hydrogel development for more complicated research studies. In this study, there are two questions without acceptable answers:

1. Numerous papers have been published on hydrogel applications and progress in cancer treatments, but it is not still clear how much these effects match with the results of a real treatment if the same study is done on an actual patient.

2. As far as we know, many of the previous studies were restricted to the models at the stage of distributed cells within the scaffold (DCS), and no study has evaluated cancer treatment and appropriate results in the next step (when the tissue is formed) in comparison with the DCS stage.

## Author contributions

The main idea and the issue for study come from Javad Esmaeili under the supervision of Dr Aboulfazl Barati and Dr Jafar Ai. All authors participated in the literature review, and also classification of the headings. All authors also participated in writing and editing the whole paper.

## Conflicts of interest

There are no financial conflicts of interest to disclose.

## Supplementary Material

## References

[cit1] Costa M. W. , Hasham M. G. and Rosenthal N., 1 - Molecular Tools in Cancer Research, in Abeloff's Clinical Oncology, J. E. Niederhuber, J. O. Armitage, M. B. Kastan, J. H. Doroshow and J. E. Tepper, Content Repository Only!, Philadelphia, 6th edn, 2020, pp. 2–23

[cit2] Moo T.-A., Sanford R., Dang C., Morrow M. (2018). Overview of Breast Cancer Therapy. PET Clinics.

[cit3] Chya-Yan L., Shen J., Murat G. (2018). Engineering 3D Hydrogels for Personalized In Vitro Human Tissue Models. Adv. Healthcare Mater..

[cit4] Soleimani S., Shamsi M., Ghazani M. A., Modarres H. P., Valente K. P., Saghafian M., Ashani M. M., Akbari M., Sanati-Nezhad A. (2018). Translational models of tumor angiogenesis: A nexus of in silico and in vitro models. Biotechnol. Adv..

[cit5] Miyake M., Hori S., Morizawa Y., Tatsumi Y., Nakai Y., Anai S., Torimoto K., Aoki K., Tanaka N., Shimada K., Konishi N., Toritsuka M., Kishimoto T., Rosser C. J., Fujimoto K. (2016). CXCL1-Mediated Interaction of Cancer Cells with Tumor-Associated Macrophages and Cancer-Associated Fibroblasts Promotes Tumor Progression in Human Bladder Cancer. Neoplasia.

[cit6] Society A. C. , Cancer facts & figures 2017, 2017

[cit7] Sarwar M. S., Niazi M. B. K., Jahan Z., Ahmad T., Hussain A. (2018). Preparation and characterization of PVA/nanocellulose/Ag nanocomposite films for antimicrobial food packaging. Carbohydr. Polym..

[cit8] Cheng M. , Chhaya M., Hintz M., Piper S., Visscher L., Schantz J. T., Wong C., Ung O., Wagels M., Hutmacher D. W., 6.25 Breast Tissue Engineering, in Comprehensive Biomaterials II, ed. P. Ducheyne, Elsevier, Oxford, 2017, pp. 435–454

[cit9] Hartner L. (2018). Chemotherapy for Oral Cancer. Dent. Clin. North Am..

[cit10] Akiyama Y., Kimura Y., Enatsu R., Mikami T., Wanibuchi M., Mikuni N. (2018). Advantages and Disadvantages of Combined Chemotherapy with Carmustine Wafer and Bevacizumab in Patients with Newly Diagnosed Glioblastoma: A Single-Institutional Experience. World Neurosurgery.

[cit11] Hamedi H., Moradi S., Hudson S. M., Tonelli A. E. (2018). Chitosan based hydrogels and their applications for drug delivery in wound dressings: A review. Carbohydr. Polym..

[cit12] Afzal S., Maswal M., Dar A. A. (2018). Rheological behavior of pH responsive composite hydrogels of chitosan and alginate: Characterization and its use in encapsulation of citral. Colloids Surf., B.

[cit13] Catoira M. C., Fusaro L., Di Francesco D., Ramella M., Boccafoschi F. (2019). Overview of natural hydrogels for regenerative medicine applications. J. Mater. Sci.: Mater. Med..

[cit14] Hecht H., Srebnik S. (2016). Structural Characterization of Sodium Alginate and Calcium Alginate. Biomacromolecules.

[cit15] Ahsan A., Tian W.-X., Farooq M. A., Khan D. H. (2020). An overview of hydrogels and their role in transdermal drug delivery. Int. J. Polym. Mater. Polym. Biomater..

[cit16] Khandan A., Jazayeri H., Fahmy M. D., Razavi M. J. B. T. E. (2017). Hydrogels.

[cit17] Saghazadeh S., Rinoldi C., Schot M., Kashaf S. S., Sharifi F., Jalilian E., Nuutila K., Giatsidis G., Mostafalu P., Derakhshandeh H., Yue K., Swieszkowski W., Memic A., Tamayol A., Khademhosseini A. (2018). Drug delivery systems and materials for wound healing applications. Adv. Drug Delivery Rev..

[cit18] Economidou S. N., Lamprou D. A., Douroumis D. (2018). 3D printing applications for transdermal drug delivery. Int. J. Pharm..

[cit19] Mahinroosta M., Jomeh Farsangi Z., Allahverdi A., Shakoori Z. (2018). Hydrogels as intelligent materials: A brief review of synthesis, properties and applications. Mater. Today Chem..

[cit20] Wu D., Xie X., Kadi A. A., Zhang Y. (2018). Photosensitive peptide hydrogels as smart materials for applications. Chin. Chem. Lett..

[cit21] Sun Y., Jensen H., Petersen N. J., Larsen S. W., Østergaard J. (2018). Concomitant monitoring of implant formation and drug release of in situ forming poly (lactide-co-glycolide acid) implants in a hydrogel matrix mimicking the subcutis using UV-vis imaging. J. Pharm. Biomed. Anal..

[cit22] Lee Y. , Ahn S. I. and Kim Y., Organs-on-Chips, Reference Module in Biomedical Sciences, Elsevier, 2018

[cit23] Ren X., Yang Q., Yang D., Liang Y., Dong J., Ren Y., Lu X., Xue L., Li L., Xu L. (2018). High-strength double network hydrogels as potential materials for artificial 3D scaffold of cell migration in vitro. Colloids Surf., A.

[cit24] Negrini N. C., Bonetti L., Contili L., Farè S. (2018). 3D printing of methylcellulose-based hydrogels. Bioprinting.

[cit25] Riahi R., Tamayol A., Shaegh S. A. M., Ghaemmaghami A. M., Dokmeci M. R., Khademhosseini A. (2015). Microfluidics for advanced drug delivery systems. Curr. Opin. Chem. Eng..

[cit26] Alam K., Iqbal M., Hasan A., Al-Maskari N. J. (2020). Rheological characterization of biological hydrogels in aqueous state. Appl. Biotechnol. Rep..

[cit27] Cuomo F., Cofelice M., Lopez F. J. P. (2019). Rheological characterization of hydrogels from alginate-based nanodispersion. Polymers.

[cit28] Tibbitt M. W., Anseth K. S. (2009). Hydrogels as extracellular matrix mimics for 3D cell culture. Biotechnol. Bioeng..

[cit29] Zhang Y. S., Khademhosseini A. (2017). Advances in engineering hydrogels. Science.

[cit30] Yang C. C., Burg K. J. (2015). Designing a tunable 3D heterocellular breast cancer tissue test system. J. Tissue Eng. Regener. Med..

[cit31] Chen Y., Gao D., Wang Y., Lin S., Jiang Y. (2018). A novel 3D breast-cancer-on-chip platform for therapeutic evaluation of drug delivery systems. Anal. Chim. Acta.

[cit32] Wang W., Song H., Zhang J., Li P., Li C., Wang C., Kong D., Zhao Q. (2015). An injectable, thermosensitive and multicompartment hydrogel for simultaneous encapsulation and independent release of a drug cocktail as an effective combination therapy platform. J. Controlled Release.

[cit33] Colley H. E., Hearnden V., Jones A. V., Weinreb P. H., Violette S. M., Macneil S., Thornhill M. H., Murdoch C. (2011). Development of tissue-engineered models of oral dysplasia and early invasive oral squamous cell carcinoma. Br. J. Cancer.

[cit34] Marrero B., Messina J. L., Heller R. (2009). Generation of a tumor spheroid in a microgravity environment as a 3D model of melanoma. In Vitro Cell. Dev. Biol.: Anim..

[cit35] Long T. J., Takeno M., Sprenger C. C., Plymate S. R., Ratner B. D. (2013). Capillary force seeding of sphere-templated hydrogels for tissue-engineered prostate cancer xenografts. Tissue Eng., Part C.

[cit36] Florczyk S. J., Liu G., Kievit F. M., Lewis A. M., Wu J. D., Zhang M. (2012). 3D porous chitosan-alginate scaffolds: a new matrix for studying prostate cancer cell-lymphocyte interactions in vitro. Adv. Healthcare Mater..

[cit37] Damiati S., Küpcü S., Peacock M., Eilenberger C., Zamzami M., Qadri I., Choudhry H., Sleytr U. B., Schuster B. (2017). Acoustic and hybrid 3D-printed electrochemical biosensors for the real-time immunodetection of liver cancer cells (HepG2). Biosens. Bioelectron..

[cit38] Gotoh Y., Ishizuka Y., Matsuura T., Niimi S. (2011). Spheroid formation and expression of liver-specific functions of human hepatocellular carcinoma-derived FLC-4 cells cultured in lactose-silk fibroin conjugate sponges. Biomacromolecules.

[cit39] Puente P. d. l., Fettig N., Luderer M. J., Jin A., Shah S., Muz B., Kapoor V., Goddu S. M., Salama N. N., Tsien C., Thotala D., Shoghi K., Rogers B., Azab A. K. (2018). Injectable Hydrogels for Localized Chemotherapy and Radiotherapy in Brain Tumors. J. Pharm. Sci..

[cit40] Dai X., Ma C., Lan Q., Xu T. (2016). 3D bioprinted glioma stem cells for brain tumor model and applications of drug susceptibility. Biofabrication.

[cit41] Hassell B. A., Goyal G., Lee E., Sontheimer-Phelps A., Levy O., Chen C. S., Ingber D. E. (2017). Human Organ Chip Models Recapitulate Orthotopic Lung Cancer Growth, Therapeutic Responses, and Tumor Dormancy In Vitro. Cell Rep..

[cit42] Ringuette Goulet C., Bernard G., Chabaud S., Couture A., Langlois A., Neveu B., Pouliot F., Bolduc S. (2017). Tissue-engineered human 3D model of bladder cancer for invasion study and drug discovery. Biomaterials.

[cit43] Wang Y. (2018). Programmable hydrogels. Biomaterials.

[cit44] Wang Y. (2018). Programmable hydrogels. Biomaterials.

[cit45] Deng K., Bellmann C., Fu Y., Rohn M., Guenther M., Gerlach G. (2018). Miniaturized force-compensated hydrogel-based pH sensors. Sens. Actuators, B.

[cit46] Zhang X., Kong M., Tian M.-p., Qu C.-c., Li J., Wang Y.-n., Sun Q.-j., Cheng X.-j., Chen X.-g. (2018). The temperature-responsive hydroxybutyl chitosan hydrogels with polydopamine coating for cell sheet transplantation. Int. J. Biol. Macromol..

[cit47] Hu J., Chen Y., Li Y., Zhou Z., Cheng Y. (2017). A thermo-degradable hydrogel with light-tunable degradation and drug release. Biomaterials.

[cit48] Cui H., Zhang H., Yu M., Yang F. (2018). Performance evaluation of electric-responsive hydrogels as draw agent in forward osmosis desalination. Desalination.

[cit49] Qu J., Zhao X., Ma P. X., Guo B. (2018). Injectable antibacterial conductive hydrogels with dual response to an electric field and pH for localized “smart” drug release. Acta Biomater..

[cit50] Graham S., Marina P. F., Blencowe A. (2019). Thermoresponsive polysaccharides and their thermoreversible physical hydrogel networks. Carbohydr. Polym..

[cit51] Lima-Tenório M. K., Tenório-Neto E. T., Guilherme M. R., Garcia F. P., Nakamura C. V., Pineda E. A. G., Rubira A. F. (2015). Water transport properties through starch-based hydrogel nanocomposites responding to both pH and a remote magnetic field. Chem. Eng. J..

[cit52] Olaru A.-M., Marin L., Morariu S., Pricope G., Pinteala M., Tartau-Mititelu L. (2018). Biocompatible chitosan based hydrogels for potential application in local tumour therapy. Carbohydr. Polym..

[cit53] Tian R., Chen J., Niu R. (2014). The development of low-molecular weight hydrogels for applications in cancer therapy. Nanoscale.

[cit54] Wang L., Neumann M., Fu T., Li W., Cheng X., Su B.-L. (2018). Porous and responsive hydrogels for cell therapy. Curr. Opin. Colloid Interface Sci..

[cit55] Ranjbari J., Mokhtarzadeh A., Alibakhshi A., Tabarzad M., Hejazi M., Ramezani M. (2018). Anti-Cancer Drug Delivery Using Carbohydrate-Based Polymers. Curr. Pharm. Des..

[cit56] Feng Z., Rao A. D., Cheng Z., Shin E. J., Moore J., Su L., Kim S. H., Wong J., Narang A., Herman J. M., McNutt T., Li D., Ding K. (2018). A dose prediction model for duodenum sparing with a biodegradable hydrogel spacer for pancreatic cancer radiotherapy. Int. J. Radiat. Oncol., Biol., Phys..

[cit57] Alexander J. J. (2018). Blood-brain barrier (BBB) and the complement landscape. Mol. Immunol..

[cit58] Modarres H. P., Janmaleki M., Novin M., Saliba J., El-Hajj F., RezayatiCharan M., Seyfoori A., Sadabadi H., Vandal M., Nguyen M. D., Hasan A., Sanati-Nezhad A. (2018). In vitro models and systems for evaluating the dynamics of drug delivery to the healthy and diseased brain. J. Controlled Release.

[cit59] Karsh L. I., Gross E. T., Pieczonka C. M., Aliotta P. J., Skomra C. J., Ponsky L. E., Nieh P. T., Han M., Hamstra D. A., Shore N. D. (2018). Absorbable Hydrogel Spacer Use in Prostate Radiotherapy: A Comprehensive Review of Phase 3 Clinical Trial Published Data. Urology.

[cit60] Fischer-Valuck B. W., Chundury A., Gay H., Bosch W., Michalski J. (2017). Hydrogel spacer distribution within the perirectal space in patients undergoing radiotherapy for prostate cancer: Impact of spacer symmetry on rectal dose reduction and the clinical consequences of hydrogel infiltration into the rectal wall. Practical Radiation Oncology.

[cit61] Dinh T.-K. T., Lee H. J., Macomber M. W., Apisarnthanarax S., Zeng J., Laramore G. E., Rengan R., Russell K. J., Chen J. J., Ellis W. J., Schade G. R., Liao J. J. (2020). Rectal Hydrogel Spacer Improves Late Gastrointestinal Toxicity Compared To Rectal Balloon Immobilization After Proton Beam Radiotherapy For Localized Prostate Cancer: A Retrospective Observational Study. Int. J. Radiat. Oncol., Biol., Phys..

[cit62] Hamstra D. A., Mariados N., Sylvester J., Shah D., Karsh L., Hudes R., Beyer D., Kurtzman S., Bogart J., Hsi R. A., Kos M., Ellis R., Logsdon M., Zimberg S., Forsythe K., Zhang H., Soffen E., Francke P., Mantz C., Rossi P., DeWeese T., Daignault-Newton S., Fischer-Valuck B. W., Chundury A., Gay H., Bosch W., Michalski J. (2017). Continued Benefit to Rectal Separation for Prostate Radiation Therapy: Final Results of a Phase III Trial. Int. J. Radiat. Oncol., Biol., Phys..

[cit63] Pinkawa M., Berneking V., Konig L., Frank D., Bretgeld M., Eble M. J. (2017). Hydrogel injection reduces rectal toxicity after radiotherapy for localized prostate cancer. Strahlenther. Onkol..

[cit64] Ogita M., Yamashita H., Sawayanagi S., Takahashi W., Nakagawa K. (2020). Efficacy of a hydrogel spacer in three-dimensional conformal radiation therapy for prostate cancer. Jpn. J. Clin. Oncol..

[cit65] Mariados N., Sylvester J., Shah D., Karsh L., Hudes R., Beyer D., Kurtzman S., Bogart J., Hsi R. A., Kos M., Ellis R., Logsdon M., Zimberg S., Forsythe K., Zhang H., Soffen E., Francke P., Mantz C., Rossi P., DeWeese T., Hamstra D. A., Bosch W., Gay H., Michalski J. (2015). Hydrogel Spacer Prospective Multicenter Randomized Controlled Pivotal Trial: Dosimetric and Clinical Effects of Perirectal Spacer Application in Men Undergoing Prostate Image Guided Intensity Modulated Radiation Therapy. Int. J. Radiat. Oncol., Biol., Phys..

[cit66] Pinkawa M., Berneking V., Schlenter M., Krenkel B., Eble M. J. (2017). Quality of Life After Radiation Therapy for Prostate Cancer With a Hydrogel Spacer: 5-Year Results. Int. J. Radiat. Oncol., Biol., Phys..

[cit67] Rao A. D., Feng Z., Shin E. J., He J., Waters K. M., Coquia S., DeJong R., Rosati L. M., Su L., Li D., Jackson J., Clark S., Schultz J., Hutchings D., Kim S. H., Hruban R. H., DeWeese T. L., Wong J., Narang A., Herman J. M., Ding K. (2017). A Novel Absorbable Radiopaque Hydrogel Spacer to Separate the Head of the Pancreas and Duodenum in Radiation Therapy for Pancreatic Cancer. Int. J. Radiat. Oncol., Biol., Phys..

[cit68] Wu H., Song L., Chen L., Zhang W., Chen Y., Zang F., Chen H., Ma M., Gu N., Zhang Y. (2018). Injectable magnetic supramolecular hydrogel with magnetocaloric liquid-conformal property prevents the post-operative recurrence in a breast cancer model. Acta Biomater..

[cit69] Kang G. D., Cheon S. H., Song S.-C. (2006). Controlled release of doxorubicin from thermosensitive poly(organophosphazene) hydrogels. Int. J. Pharm..

[cit70] Langer R., Tirrell D. A. (2004). Designing materials for biology and medicine. Nature.

[cit71] Ulijn R. V., Bibi N., Jayawarna V., Thornton P. D., Todd S. J., Mart R. J., Smith A. M., Gough J. E. (2007). Bioresponsive hydrogels. Mater. Today.

[cit72] Bastiancich C., Bozzato E., Luyten U., Danhier F., Bastiat G., Préat V. (2019). Drug combination using an injectable nanomedicine hydrogel for glioblastoma treatment. Int. J. Pharm..

[cit73] Peng D., Gao H., Huang P., Shi X., Zhou J., Zhang J., Dong A., Tang H., Wang W., Deng L. (2019). Host-guest supramolecular hydrogel based on nanoparticles: co-delivery of DOX and siBcl-2 for synergistic cancer therapy. J. Biomater. Sci., Polym. Ed..

[cit74] Norouzi M., Nazari B., Miller D. W. (2016). Injectable hydrogel-based drug delivery systems for local cancer therapy. Drug Discovery Today.

[cit75] Samadi A. K., Bilsland A., Georgakilas A. G., Amedei A., Amin A., Bishayee A., Azmi A. S., Lokeshwar B. L., Grue B., Panis C., Boosani C. S., Poudyal D., Stafforini D. M., Bhakta D., Niccolai E., Guha G., Vasantha Rupasinghe H. P., Fujii H., Honoki K., Mehta K., Aquilano K., Lowe L., Hofseth L. J., Ricciardiello L., Ciriolo M. R., Singh N., Whelan R. L., Chaturvedi R., Ashraf S. S., Shantha Kumara H. M. C., Nowsheen S., Mohammed S. I., Keith W. N., Helferich W. G., Yang X. (2015). A multi-targeted approach to suppress tumor-promoting inflammation. Semin. Cancer Biol..

[cit76] Coussens L. M., Werb Z. (2002). Inflammation and cancer. Nature.

[cit77] Tao M., He S., Liu J., Li H., Mei L., Wu C., Xu K., Zhong W. (2019). The conjugates of forky peptides and nonsteroidal anti-inflammatory drugs (NSAID) self-assemble into supramolecular hydrogels for prostate cancer-specific drug delivery. J. Mater. Chem. B.

[cit78] Zhang Y., Sun T., Jiang C. (2018). Biomacromolecules as carriers in drug delivery and tissue engineering. Acta Pharm. Sin. B.

[cit79] Mills J. K., Needham D. (1999). Targeted drug delivery. Expert Opin. Ther. Pat..

[cit80] Ta H. T., Dass C. R., Dunstan D. E. (2008). Injectable chitosan hydrogels for localised cancer therapy. J. Controlled Release.

[cit81] Kennedy P. J., Oliveira C., Granja P. L., Sarmento B. (2017). Antibodies and associates: Partners in targeted drug delivery. Pharmacol. Ther..

[cit82] Guziewicz N., Best A., Perez-Ramirez B., Kaplan D. L. (2011). Lyophilized silk fibroin hydrogels for the sustained local delivery of therapeutic monoclonal antibodies. Biomaterials.

[cit83] Fernandes E., Ferreira J. A., Andreia P., Luís L., Barroso S., Sarmento B., Santos L. L. (2015). New trends in guided nanotherapies for digestive cancers: A systematic review. J. Controlled Release.

[cit84] Ishihara M., Obara K., Nakamura S., Fujita M., Masuoka K., Kanatani Y., Takase B., Hattori H., Morimoto Y., Ishihara M., Maehara T., Kikuchi M. (2006). Chitosan hydrogel as a drug delivery carrier to control angiogenesis. J. Artif. Organs.

[cit85] Jamal A., Shahzadi L., Ahtzaz S., Zahid S., Chaudhry A. A., Rehman I. u., Yar M. (2018). Identification of anti-cancer potential of doxazocin: Loading into chitosan based biodegradable hydrogels for on-site
delivery to treat cervical cancer. Mater. Sci. Eng., C.

[cit86] Karavasili C., Andreadis D. A., Katsamenis O. L., Panteris E., Anastasiadou P., Kakazanis Z., Zoumpourlis V., Markopoulou C. K., Koutsopoulos S., Vizirianakis I. S., Fatouros D. G. (2019). Synergistic Antitumor Potency of a Self-Assembling Peptide Hydrogel for the Local Co-delivery of Doxorubicin and Curcumin in the Treatment of Head and Neck Cancer. Mol. Pharm..

[cit87] Shu C., Li R., Yin Y., Yin D., Gu Y., Ding L., Zhong W. (2014). Synergistic dual-targeting hydrogel improves targeting and anticancer effect of Taxol in vitro and in vivo. Chem. Commun..

[cit88] Xing F., Saidou J., Watabe K. (2010). Cancer associated fibroblasts (CAFs) in tumor microenvironment. Front. Biosci., Landmark Ed..

[cit89] Liu M., Song W., Huang L. (2019). Drug delivery systems targeting tumor-associated fibroblasts for cancer immunotherapy. Cancer Lett..

[cit90] Hu C., Liu X., Ran W., Meng J., Zhai Y., Zhang P., Yin Q., Yu H., Zhang Z., Li Y. (2017). Regulating cancer associated fibroblasts with losartan-loaded injectable peptide hydrogel to potentiate chemotherapy in inhibiting growth and lung metastasis of triple negative breast cancer. Biomaterials.

[cit91] Zhao D., Song H., Zhou X., Chen Y., Liu Q., Gao X., Zhu X., Chen D. (2019). Novel facile thermosensitive hydrogel as sustained and controllable gene release vehicle for breast cancer treatment. Eur. J. Pharm. Sci..

[cit92] Huang G., Huang H. (2018). Hyaluronic acid-based biopharmaceutical delivery and tumor-targeted drug delivery system. J. Controlled Release.

[cit93] Zhang W., Cheng Q., Guo S., Lin D., Huang P., Liu J., Wei T., Deng L., Liang Z., Liang X. J., Dong A. (2013). Gene transfection efficacy and biocompatibility of polycation/DNA complexes coated with enzyme degradable PEGylated hyaluronic acid. Biomaterials.

[cit94] Wang X., Gu X., Wang H., Yang J., Mao S. (2018). Enhanced delivery of doxorubicin to the liver through self-assembled nanoparticles formed via conjugation of glycyrrhetinic acid to the hydroxyl group of hyaluronic acid. Carbohydr. Polym..

[cit95] Chen X., Liu Z., Parker S. G., Zhang X., Gooding J. J., Ru Y., Liu Y., Zhou Y. (2016). Light-Induced Hydrogel Based on Tumor-Targeting Mesoporous Silica Nanoparticles as a Theranostic Platform for Sustained Cancer Treatment. ACS Appl. Mater. Interfaces.

[cit96] Cacicedo M. L., Islan G. A., León I. E., Álvarez V. A., Chourpa I., Allard-Vannier E., García-Aranda N., Díaz-Riascos Z. V., Fernández Y., Schwartz S., Abasolo I., Castro G. R. (2018). Bacterial cellulose hydrogel loaded with lipid nanoparticles for localized cancer treatment. Colloids Surf., B.

[cit97] Carvalho S. M., Mansur A. A. P., Capanema N. S. V., Carvalho I. C., Chagas P., de Oliveira L. C. A., Mansur H. S. (2018). Synthesis and in vitro assessment of anticancer hydrogels composed by carboxymethylcellulose-doxorubicin as potential transdermal delivery systems for treatment of skin cancer. J. Mol. Liq..

[cit98] Rammohan A., Sathyanesan J., Ramaswami S., Lakshmanan A., Senthil-Kumar P., Srinivasan U. P., Ramasamy R., Ravichandran P. (2012). Embolization of liver tumors: Past, present and future. World J. Radiol..

[cit99] Poursaid A., Jensen M. M., Huo E., Ghandehari H. (2016). Polymeric materials for embolic and chemoembolic applications. J. Controlled Release.

[cit100] Zhou X., Li Y., Chen S., Fu Y.-n., Wang S., Li G., Tao L., Wei Y., Wang X., Liang J. F. (2018). Dynamic agent of an injectable and self-healing drug-loaded hydrogel for embolization therapy. Colloids Surf., B.

[cit101] Singh N. K., Lee D. S. (2014). In situ gelling pH- and temperature-sensitive biodegradable block copolymer hydrogels for drug delivery. J. Controlled Release.

[cit102] Sood N., Bhardwaj A., Mehta S., Mehta A. (2016). Stimuli-responsive hydrogels in drug delivery and tissue engineering. Drug Delivery.

[cit103] Davaran S., Ghamkhari A., Alizadeh E., Massoumi B., Jaymand M. (2017). Novel dual stimuli-responsive ABC triblock copolymer: RAFT synthesis, "schizophrenic" micellization, and its performance as an anticancer drug delivery nanosystem. J. Colloid Interface Sci..

[cit104] Carlin K. (2014). Autoimmune Disease pH and Temperature. J. Clin. Med. Res..

[cit105] Swietach P., Vaughan-Jones R. D., Harris A. L., Hulikova A. (2014). The chemistry, physiology and pathology of pH in cancer. Philos. Trans. R. Soc. London, Ser. B.

[cit106] Liu J., Huang Y., Kumar A., Tan A., Jin S., Mozhi A., Liang X.-J. (2014). pH-Sensitive nano-systems for drug delivery in cancer therapy. Biotechnol. Adv..

[cit107] Narkhede A. A., Crenshaw J. H., Manning R. M., Rao S. S. (2018). The influence of matrix stiffness on the behavior of brain metastatic breast cancer cells in a biomimetic hyaluronic acid hydrogel platform. J. Biomed. Mater. Res., Part A.

[cit108] Wu X., He C., Wu Y., Chen X. (2016). Synergistic therapeutic effects of Schiff's base cross-linked injectable hydrogels for local co-delivery of metformin and 5-fluorouracil in a mouse colon carcinoma model. Biomaterials.

[cit109] Nagahama K., Kawano D., Oyama N., Takemoto A., Kumano T., Kawakami J. (2015). Self-assembling polymer micelle/clay nanodisk/doxorubicin hybrid injectable gels for safe and efficient focal treatment of cancer. Biomacromolecules.

[cit110] Song J., Hwang S., Im K., Hur J., Nam J., Hwang S., Ahn G. O., Kim S., Park N. (2015). Light-responsible DNA hydrogel–gold nanoparticle assembly for synergistic cancer therapy. J. Mater. Chem. B.

[cit111] Steichen S. D., Caldorera-Moore M., Peppas N. A. (2013). A review of current nanoparticle and targeting moieties for the delivery of cancer therapeutics. Eur. J. Pharm. Sci..

[cit112] Mao Y., Li X., Chen G., Wang S. (2016). Thermosensitive Hydrogel System With Paclitaxel Liposomes Used in Localized Drug Delivery System for In Situ Treatment of Tumor: Better Antitumor Efficacy and Lower Toxicity. J. Pharm. Sci..

[cit113] Guo D. D., Hong S. H., Jiang H. L., Kim J. H., Minai-Tehrani A., Kim J. E., Shin J. Y., Jiang T., Kim Y. K., Choi Y. J., Cho C. S., Cho M. H. (2012). Synergistic effects of Akt1 shRNA and paclitaxel-incorporated conjugated linoleic acid-coupled poloxamer thermosensitive hydrogel on breast cancer. Biomaterials.

[cit114] Wu Z., Zou X., Yang L., Lin S., Fan J., Yang B., Sun X., Wan Q., Chen Y., Fu S. (2014). Thermosensitive hydrogel used in dual drug delivery system with paclitaxel-loaded micelles for in situ treatment of lung cancer. Colloids Surf., B.

[cit115] Zhu X., Zhang Y., Huang H., Zhang H., Hou L., Zhang Z. (2016). Functionalized graphene oxide-based thermosensitive hydrogel for near-infrared chemo-photothermal therapy on tumor. J. Biomater. Appl..

[cit116] Du H., Liu M., Yang X., Zhai G. (2015). The design of pH-sensitive chitosan-based formulations for gastrointestinal delivery. Drug Discovery Today.

[cit117] Qian C., Zhang T., Gravesande J., Baysah C., Song X., Xing J. (2019). Injectable and self-healing polysaccharide-based hydrogel for pH-responsive drug release. Int. J. Biol. Macromol..

[cit118] Ding L., Li J., Wu C., Yan F., Li X., Zhang S. (2020). A self-assembled RNA-triple helix hydrogel drug delivery system targeting triple-negative breast cancer. J. Mater. Chem. B.

[cit119] Yang H., Tang C., Yin C. (2018). Estrone-modified pH-sensitive glycol chitosan nanoparticles for drug delivery in breast cancer. Acta Biomater..

[cit120] Wang Z., Deng X., Ding J., Zhou W., Zheng X., Tang G. (2018). Mechanisms of drug release in pH-sensitive micelles for tumour targeted drug delivery system: A review. Int. J. Pharm..

[cit121] Almurshedi A. S., Radwan M., Omar S., Alaiya A. A., Badran M. M., Elsaghire H., Saleem I. Y., Hutcheon G. A. (2018). A novel pH-sensitive liposome to trigger delivery of afatinib to cancer cells: Impact on lung cancer therapy. J. Mol. Liq..

[cit122] Wu W., Shen J., Banerjee P., Zhou S. (2010). Chitosan-based responsive hybrid nanogels for integration of optical pH-sensing, tumor cell imaging and controlled drug delivery. Biomaterials.

[cit123] Xiong W., Wang W., Wang Y., Zhao Y., Chen H., Xu H., Yang X. (2011). Dual temperature/pH-sensitive drug delivery of poly(N-isopropylacrylamide-co-acrylic acid) nanogels conjugated with doxorubicin for potential application in tumor hyperthermia therapy. Colloids Surf., B.

[cit124] Repasky E. A., Evans S. S., Dewhirst M. W. (2013). Temperature matters! And why it should matter to tumor immunologists. Cancer Immunol. Res..

[cit125] Sponchioni M., Capasso Palmiero U., Moscatelli D. (2019). Thermo-responsive polymers: Applications of smart materials in drug delivery and tissue engineering. Mater. Sci. Eng., C.

[cit126] An H., Xu K., Chang L., Wang Y., Qin J., Li W. (2018). Thermo-responsive self-healable hydrogels with extremely mild base degradability and bio-compatibility. Polymer.

[cit127] Lv Q., He C., Quan F., Yu S., Chen X. (2018). DOX/IL-2/IFN-γ co-loaded thermo-sensitive polypeptide hydrogel for efficient melanoma treatment. Bioact. Mater..

[cit128] Indulekha S., Arunkumar P., Bahadur D., Srivastava R. (2017). Dual responsive magnetic composite nanogels for thermo-chemotherapy. Colloids Surf., B.

[cit129] Moreira A. F., Dias D. R., Costa E. C., Correia I. J. (2017). Thermo- and pH-responsive nano-in-micro particles for combinatorial drug delivery to cancer cells. Eur. J. Pharm. Sci..

[cit130] Shi W., Ji Y., Zhang X., Shu S., Wu Z. (2011). Characterization of ph- and thermosensitive hydrogel as a vehicle for controlled protein delivery. J. Pharm. Sci..

[cit131] Bostan M. S., Senol M., Cig T., Peker I., Goren A. C., Ozturk T., Eroglu M. S. (2013). Controlled release of 5-aminosalicylicacid from chitosan based pH and temperature sensitive hydrogels. Int. J. Biol. Macromol..

[cit132] Fong Y. T., Chen C. H., Chen J. P. (2017). Intratumoral Delivery of Doxorubicin on Folate-Conjugated Graphene Oxide by In-Situ Forming Thermo-Sensitive Hydrogel for Breast Cancer Therapy. Nanomaterials.

[cit133] Zhang W., Jin X., Li H., Zhang R.-r., Wu C.-w. (2018). Injectable and body temperature sensitive hydrogels based on chitosan and hyaluronic acid for pH sensitive drug release. Carbohydr. Polym..

[cit134] Wu J., Xie X., Zheng Z., Li G., Wang X., Wang Y. (2017). Effect of pH on polyethylene glycol (PEG)-induced silk microsphere formation for drug delivery. Mater. Sci. Eng., C.

[cit135] Xie J., Yang Z., Zhou C., Zhu J., Lee R. J., Teng L. (2016). Nanotechnology for the delivery of phytochemicals in cancer therapy. Biotechnol. Adv..

[cit136] Jeetah R., Bhaw-Luximon A., Jhurry D. (2014). Polymeric nanomicelles for sustained delivery of anti-cancer drugs. Mutat. Res., Fundam. Mol. Mech. Mutagen..

[cit137] Phan V. H. G., Thambi T., Duong H. T. T., Lee D. S. (2016). Poly(amino carbonate urethane)-based biodegradable, temperature and pH-sensitive injectable hydrogels for sustained human growth hormone delivery. Sci. Rep..

[cit138] Bolla P. K., Rodriguez V. A., Kalhapure R. S., Kolli C. S., Andrews S., Renukuntla J. (2018). A review on pH and temperature responsive gels and other less explored drug delivery systems. J. Drug Delivery Sci. Technol..

[cit139] Plamper F. A., Richtering W. (2017). Functional Microgels and Microgel Systems. Acc. Chem. Res..

[cit140] Li Z., Ngai T. (2013). Microgel particles at the fluid–fluid interfaces. Nanoscale.

[cit141] Zhang Z.-Q., Song S.-C. (2016). Thermosensitive/superparamagnetic iron oxide nanoparticle-loaded nanocapsule hydrogels for multiple cancer hyperthermia. Biomaterials.

[cit142] Spang M. T., Christman K. L. (2018). Extracellular matrix hydrogel therapies: In vivo applications and development. Acta Biomater..

[cit143] Gadde M. , Marrinan D., Michna R. J., Rylander M. N., Three Dimensional In Vitro Tumor Platforms for Cancer Discovery, in Tumor Organoids, ed. S. Soker and A. Skardal, Springer International Publishing, Cham, 2018, pp. 71–94

[cit144] Brancato V., Oliveira J. M., Correlo V. M., Reis R. L., Kundu S. C. (2020). Could 3D models of cancer enhance drug screening?. Biomaterials.

[cit145] Gonzalez Gonzalez M., Cichon I., Scislowska-Czarnecka A., Kolaczkowska E. (2018). Challenges in 3D culturing of neutrophils: Assessment of cell viability. J. Immunol. Methods.

[cit146] Yue X., Nguyen T. D., Zellmer V., Zhang S., Zorlutuna P. (2018). Stromal cell-laden 3D hydrogel microwell arrays as tumor microenvironment model for studying stiffness dependent stromal cell-cancer interactions. Biomaterials.

[cit147] Brancato V., Gioiella F., Imparato G., Guarnieri D., Urciuolo F., Netti P. A. (2018). 3D breast cancer microtissue reveals the role of tumor microenvironment on the transport and efficacy of free-doxorubicin in vitro. Acta Biomater..

[cit148] Xin X., Yang H., Zhang F., Yang S.-T. (2019). 3D cell coculture tumor model: A promising approach for future cancer drug discovery. Process Biochem..

[cit149] Shiga K., Hara M., Nagasaki T., Sato T., Takahashi H., Takeyama H. (2015). Cancer-Associated Fibroblasts: Their Characteristics and Their Roles in Tumor Growth. Cancers.

[cit150] Rodenhizer D., Cojocari D., Wouters B. G., McGuigan A. P. (2016). Development of TRACER: tissue roll for analysis of cellular environment and response. Biofabrication.

[cit151] Young M., Rodenhizer D., Dean T., D'Arcangelo E., Xu B., Ailles L., McGuigan A. P. (2018). A TRACER 3D Co-Culture tumour model for head and neck cancer. Biomaterials.

[cit152] Sheikholeslam M., Wheeler S. D., Duke K. G., Marsden M., Pritzker M., Chen P. (2018). Peptide and peptide-carbon nanotube hydrogels as scaffolds for tissue & 3D tumor engineering. Acta Biomater..

[cit153] Tan T., Wang Y., Wang J., Wang Z., Wang H., Cao H., Li J., Li Y., Zhang Z., Wang S. (2019). Targeting peptide-decorated biomimetic lipoproteins improve deep penetration and cancer cells accessibility in solid tumor. Acta Pharm. Sin. B.

[cit154] Khurana A. and Godugu C., Alginate-Based Three-Dimensional In Vitro Tumor Models: A Better Alternative to Current Two-Dimensional Cell Culture Models, in Alginates and Their Biomedical Applications, B. H. A. Rehm and M. F. Moradali, Springer Singapore, Singapore, 2018, pp. 157–183

[cit155] Mennerich D., Kubaichuk K., Kietzmann T. (2019). DUBs, Hypoxia, and Cancer. Trends Cancer.

[cit156] Lewis D. M., Gerecht S., Eisinger-Mathason T. S. K. (2017). Abstract 5477: Hydrogels to study ECM-oxygen gradient interactions for sarcoma cell migration. Cancer Res..

[cit157] Liu Q., Zhang Z., Liu Y., Cui Z., Zhang T., Li Z., Ma W. (2018). Cancer cells growing on perfused 3D collagen model produced higher reactive oxygen species level and were more resistant to cisplatin compared to the 2D model. J. Appl. Biomater. Funct. Mater..

[cit158] Liu C., Lewin Mejia D., Chiang B., Luker K. E., Luker G. D. (2018). Hybrid collagen alginate hydrogel as a platform for 3D tumor spheroid invasion. Acta Biomater..

[cit159] Combellack E. J., Jessop Z. M., Naderi N., Griffin M., Dobbs T., Ibrahim A., Evans S., Burnell S., Doak S. H., Whitaker I. S. (2016). Adipose regeneration and implications for breast reconstruction: update and the future. Gland Surg..

[cit160] Hume R. D., Berry L., Reichelt S., D’Angelo M., Gomm J., Cameron R. E., Watson C. J. (2018). An Engineered Human Adipose/Collagen Model for In Vitro Breast Cancer Cell Migration Studies. Tissue Eng., Part A.

[cit161] Cao H., Lee M. K. H., Yang H., Sze S. K., Tan N. S., Tay C. Y. (2018). Mechanoregulation of cancer-associated fibroblasts phenotype in 3D interpenetrating hydrogel networks. Langmuir.

[cit162] Suo A., Xu W., Wang Y., Sun T., Ji L., Qian J. (2019). Dual-degradable and injectable hyaluronic acid hydrogel mimicking extracellular matrix for 3D culture of breast cancer MCF-7 cells. Carbohydr. Polym..

[cit163] White E. A., Kenny H. A., Lengyel E. (2014). Three-dimensional modeling of ovarian cancer. Adv. Drug Delivery Rev..

[cit164] Wang X., Zhang X., Dai X., Wang X., Li X., Diao J., Xu T. (2018). Tumor-like lung cancer model based on 3D bioprinting. 3 Biotech.

[cit165] Read G. H., Miura N., Carter J. L., Kines K. T., Yamamoto K., Devasahayam N., Cheng J. Y., Camphausen K. A., Krishna M. C., Kesarwala A. H. (2018). Three-dimensional alginate hydrogels for radiobiological and metabolic studies of cancer cells. Colloids Surf., B.

[cit166] Lin C.-C., Korc M. (2018). Designer hydrogels: Shedding light on the physical chemistry of the pancreatic cancer microenvironment. Cancer Lett..

[cit167] De Luca A., Raimondi L., Salamanna F., Carina V., Costa V., Bellavia D., Alessandro R., Fini M., Giavaresi G. (2018). Relevance of 3d culture systems to study osteosarcoma environment. J. Exp. Clin. Cancer Res..

[cit168] Ta H. T., Dass C. R., Larson I., Choong P. F., Dunstan D. E. (2009). A chitosan hydrogel delivery system for osteosarcoma gene therapy with pigment epithelium-derived factor combined with chemotherapy. Biomaterials.

[cit169] Song H.-H. G., Park K. M., Gerecht S. (2014). Hydrogels to model 3D in vitro microenvironment of tumor vascularization. Adv. Drug Delivery Rev..

[cit170] Kalkal A. , Ahmad N., Gopinath P. and Vinogradov A., Chapter 1 - 3D Printing in Medicine: Current Challenges and Potential Applications, in 3D Printing Technology in Nanomedicine, Elsevier, 2019, pp. 1–22

[cit171] Das P., Colombo M., Prosperi D. (2019). Recent advances in magnetic fluid hyperthermia for cancer therapy. Colloids Surf., B.

[cit172] Zahedi-Tabar Z., Bagheri-Khoulenjani S., Mirzadeh H., Amanpour S. (2019). 3D in vitro cancerous tumor models: Using 3D printers. Med. Hypotheses.

[cit173] Almela T., Al-Sahaf S., Brook I. M., Khoshroo K., Rasoulianboroujeni M., Fahimipour F., Tahriri M., Dashtimoghadam E., Bolt R., Tayebi L., Moharamzadeh K. (2018). 3D printed tissue engineered model for bone invasion of oral cancer. Tissue Cell.

[cit174] Rodrigues T., Kundu B., Silva-Correia J., Kundu S. C., Oliveira J. M., Reis R. L., Correlo V. M. (2018). Emerging tumor spheroids technologies for 3D in vitro cancer modeling. Pharmacol. Ther..

[cit175] Costa E. C., Moreira A. F., de Melo-Diogo D., Gaspar V. M., Carvalho M. P., Correia I. J. (2016). 3D tumor spheroids: an overview on the tools and techniques used for their analysis. Biotechnol. Adv..

[cit176] Jiang T., Munguia-Lopez J., Flores-Torres S., Grant J., Vijayakumar S., De Leon-Rodriguez A., Kinsella J. M. (2018). Bioprintable Alginate/Gelatin Hydrogel 3D In Vitro Model Systems Induce Cell Spheroid Formation. J. Visualized Exp..

[cit177] Luo Y., Wei X., Wan Y., Lin X., Wang Z., Huang P. (2019). 3D printing of hydrogel scaffolds for future application in photothermal therapy of breast cancer and tissue repair. Acta Biomater..

[cit178] Jogalekar M. P., Serrano E. E. (2018). Morphometric analysis of a triple negative breast cancer cell line in hydrogel and monolayer culture environments. PeerJ.

[cit179] Santos L. F., Correia I. J., Silva A. S., Mano J. F. (2018). Biomaterials for drug delivery patches. Eur. J. Pharm. Sci..

[cit180] Pere C. P. P., Economidou S. N., Lall G., Ziraud C., Boateng J. S., Alexander B. D., Lamprou D. A., Douroumis D. (2018). 3D printed microneedles for insulin skin delivery. Int. J. Pharm..

[cit181] Ballard D. H., Trace A. P., Ali S., Hodgdon T., Zygmont M. E., DeBenedectis C. M., Smith S. E., Richardson M. L., Patel M. J., Decker S. J., Lenchik L. (2018). Clinical Applications of 3D Printing: Primer for Radiologists. Acad. Radiol..

[cit182] Xi H., Yang Y., Zhao D., Fang L., Sun L., Mu L., Liu J., Zhao N., Zhao Y., Zheng N., He Z. (2010). Transdermal patches for site-specific delivery of anastrozole: In vitro and local tissue disposition evaluation. Int. J. Pharm..

[cit183] Kong B. J., Kim A., Park S. N. (2016). Properties and in vitro drug release of hyaluronic acid-hydroxyethyl cellulose hydrogels for transdermal delivery of isoliquiritigenin. Carbohydr. Polym..

[cit184] Sharma A., Sasaki D., Rickey D. W., Leylek A., Harris C., Johnson K., Alpuche Aviles J. E., McCurdy B., Egtberts A., Koul R., Dubey A. (2018). Low-cost optical scanner and 3-dimensional printing technology to create lead shielding for radiation therapy of facial skin cancer: First clinical case series. Advances in Radiation Oncology.

[cit185] Yoon S. H., Park S., Kang C. H., Park I. K., Goo J. M., Kim Y. T. (2018). Personalized 3D-Printed Model for Informed Consent for Stage I Lung Cancer: A Randomized Pilot Trial. Semin. Thorac. Cardiovasc. Surg..

[cit186] Mou H., Wang J., Hu H., Xu W., Chen Q. (2015). Non-small cell lung cancer 95D cells co-cultured with 3D-bioprinted scaffold to construct a lung cancer model in vitro. Zhonghua Zhongliu Zazhi.

[cit187] Zhang H., Liu G., Tong X. G., Hang W. (2018). Application of three-dimensional printing technology in the surgical treatment of nasal skull base tumor. Zhonghua Er Bi Yan Hou Tou Jing Wai Ke Za Zhi.

[cit188] Ahangar P., Akoury E., Ramirez Garcia Luna A. S., Nour A., Weber M. H., Rosenzweig D. H. (2018). Nanoporous 3D-Printed Scaffolds for Local Doxorubicin Delivery in Bone Metastases Secondary to Prostate Cancer. Materials.

[cit189] Park J. W., Kang H. G., Lim K. M., Park D. W., Kim J. H., Kim H. S. (2018). Bone tumor resection guide using three-dimensional printing for limb salvage surgery. J. Surg. Oncol..

[cit190] Zhong J., Zhang Y., Chen J., Huang R., Yang Y., Chen H., Huang Y., Tan W., Tan Z. (2018). In Vitro Study of Colon Cancer Cell Migration Using E-Jet 3D Printed Cell Culture Platforms. Macromol. Biosci..

[cit191] Dupret-Bories A., Vergez S., Meresse T., Brouillet F., Bertrand G. (2018). Contribution of 3D printing to mandibular reconstruction after cancer. European Annals of Otorhinolaryngology, Head and Neck Diseases.

[cit192] Pauls J. P. , Bartnikowski N., Jansen S.-H., Lim E. and Dasse K., Chapter 13 - Preclinical evaluation, in Mechanical Circulatory and Respiratory Support, ed. S. D. Gregory, M. C. Stevens and J. F. Fraser, Academic Press, 2018, pp. 407–438

[cit193] Ronaldson-Bouchard K., Vunjak-Novakovic G. (2018). Organs-on-a-Chip: A Fast Track for Engineered Human Tissues in Drug Development. Cell Stem Cell.

[cit194] Bein A., Shin W., Jalili-Firoozinezhad S., Park M. H., Sontheimer-Phelps A., Tovaglieri A., Chalkiadaki A., Kim H. J., Ingber D. E. (2018). Microfluidic Organ-on-a-Chip Models of Human Intestine. Cellular and Molecular Gastroenterology and Hepatology.

[cit195] Irimia D., Wang X. (2018). Inflammation-on-a-Chip: Probing the Immune System Ex Vivo. Trends Biotechnol..

[cit196] Sriram G., Alberti M., Dancik Y., Wu B., Wu R., Feng Z., Ramasamy S., Bigliardi P. L., Bigliardi-Qi M., Wang Z. (2018). Full-thickness human skin-on-chip with enhanced epidermal morphogenesis and barrier function. Mater. Today.

[cit197] Beckwitt C. H., Clark A. M., Wheeler S., Taylor D. L., Stolz D. B., Griffith L., Wells A. (2018). Liver ‘organ on a chip’. Exp. Cell Res..

[cit198] Lee J. , Kim K. and Kim S., Kidney on chips, Methods in Cell Biology, Academic Press, 2018 10.1016/bs.mcb.2018.06.001 30037468

[cit199] Biselli E., Agliari E., Barra A., Bertani F. R., Gerardino A., De Ninno A., Mencattini A., Di Giuseppe D., Mattei F., Schiavoni G., Lucarini V., Vacchelli E., Kroemer G., Di Natale C., Martinelli E., Businaro L. (2017). Organs on chip approach: a tool to evaluate cancer -immune cells interactions. Sci. Rep..

[cit200] Shukla V. C., Kuang T.-r., Senthilvelan A., Higuita-Castro N., Duarte-Sanmiguel S., Ghadiali S. N., Gallego-Perez D. (2018). Lab-on-a-Chip Platforms for Biophysical Studies of Cancer with Single-Cell Resolution. Trends Biotechnol..

[cit201] Jie M. and Lin J.-M., Microfluidic Cell Culture Systems for Drug Research, in Cell Analysis on Microfluidics, ed. J.-M. Lin, Springer Singapore, Singapore, 2018, pp. 339–370

[cit202] Kimura H., Sakai Y., Fujii T. (2018). Organ/body-on-a-chip based on microfluidic technology for drug discovery. Drug Metab. Pharmacokinet..

[cit203] Lee Y. , Ahn S. I. and Kim Y., Organs-on-Chips, in Encyclopedia of Biomedical Engineering, ed. R. Narayan, Elsevier, Oxford, 2019, pp. 384–393

[cit204] Liu W., Song J., Du X., Zhou Y., Li Y., Li R., Lyu L., He Y., Hao J., Ben J., Wang W., Shi H., Wang Q. (2019). AKR1B10 (Aldo-keto reductase family 1 B10) promotes brain metastasis of lung cancer cells in a multi-organ microfluidic chip model. Acta Biomater..

[cit205] Sanjay S. T., Zhou W., Dou M., Tavakoli H., Ma L., Xu F., Li X. (2018). Recent advances of controlled drug delivery using microfluidic platforms. Adv. Drug Delivery Rev..

[cit206] Orcheston-Findlay L., Hashemi A., Garrill A., Nock V. (2018). A microfluidic gradient generator to simulate the oxygen microenvironment in cancer cell culture. Microelectron. Eng..

[cit207] Hassell B. A., Goyal G., Lee E., Sontheimer-Phelps A., Levy O., Chen C. S., Ingber D. E. (2018). Human Organ Chip Models Recapitulate Orthotopic Lung Cancer Growth, Therapeutic Responses, and Tumor Dormancy In Vitro. Cell Rep..

[cit208] Fan Q., Liu R., Jiao Y., Tian C., Farrell J. D., Diao W., Wang X., Zhang F., Yuan W., Han H., Chen J., Yang Y., Zhang X., Ye F., Li M., Ouyang Z., Liu L. (2017). A novel 3-D bio-microfluidic system mimicking in vivo heterogeneous tumour microstructures reveals complex tumour–stroma interactions. Lab Chip.

[cit209] Filomena G., Francesco U., Giorgia I., Virginia B., Netti P. A. (2016). An Engineered Breast Cancer Model on a Chip to Replicate ECM-Activation In Vitro during Tumor Progression. Adv. Healthcare Mater..

[cit210] Eslami Amirabadi H., SahebAli S., Frimat J. P., Luttge R., den Toonder J. M. J. (2017). A novel method to understand tumor cell invasion: integrating extracellular matrix mimicking layers in microfluidic chips by “selective curing”. Biomed. Microdevices.

[cit211] Chen Y., Gao D., Liu H., Lin S., Jiang Y. (2015). Drug cytotoxicity and signaling pathway analysis with three-dimensional tumor spheroids in a microwell-based microfluidic chip for drug screening. Anal. Chim. Acta.

[cit212] Aleman J., Skardal A. (2018). A multi-site metastasis-on-a-chip microphysiological system for assessing metastatic preference of cancer cells. Biotechnol. Bioeng..

[cit213] Caballero D., Kaushik S., Correlo V. M., Oliveira J. M., Reis R. L., Kundu S. C. (2017). Organ-on-chip models of cancer metastasis for future personalized medicine: From chip to the patient. Biomaterials.

[cit214] Lee E., Song H. H. G., Chen C. S. (2016). Biomimetic on-a-chip platforms for studying cancer metastasis. Curr. Opin. Chem. Eng..

[cit215] Skardal A., Devarasetty M., Forsythe S., Atala A., Soker S. (2016). A reductionist metastasis-on-a-chip platform for in vitro tumor progression modeling and drug screening. Biotechnol. Bioeng..

[cit216] Sun Y., Haglund T. A., Rogers A. J., Ghanim A. F., Sethu P. (2018). Review: Microfluidics technologies for blood-based cancer liquid biopsies. Anal. Chim. Acta.

[cit217] Chen Y., Gao D., Wang Y., Lin S., Jiang Y. (2018). A novel 3D breast-cancer-on-chip platform for therapeutic evaluation of drug delivery systems. Anal. Chim. Acta.

[cit218] Lewis D. M., Gerecht S. (2016). Microfluidics and biomaterials to study angiogenesis. Curr. Opin. Chem. Eng..

[cit219] Lu S., Cuzzucoli F., Jiang J., Liang L.-G., Wang Y., Kong M., Zhao X., Cui W., Li J., Wang S. (2018). Development of a biomimetic liver tumor-on-a-chip model based on decellularized liver matrix for toxicity testing. Lab Chip.

[cit220] Hassell B. A., Goyal G., Lee E., Sontheimer-Phelps A., Levy O., Chen C. S., Ingber D. E. (2017). Human Organ Chip Models Recapitulate Orthotopic Lung Cancer Growth, Therapeutic Responses, and Tumor Dormancy In Vitro. Cell Rep..

[cit221] Hao S., Ha L., Cheng G., Wan Y., Xia Y., Sosnoski D. M., Mastro A. M., Zheng S. Y. (2018). A Spontaneous 3D Bone-On-a-Chip for Bone Metastasis Study of Breast Cancer Cells. Small.

[cit222] Sepantafar M., Maheronnaghsh R., Mohammadi H., Radmanesh F., Hasani-sadrabadi M. M., Ebrahimi M., Baharvand H. (2017). Engineered Hydrogels in Cancer Therapy and Diagnosis. Trends Biotechnol..

